# Serum Extracellular Vesicles as Pathogenetic Signals in Obese and Lean Patients with Metabolic Dysfunction-Associated Steatotic Liver Disease

**DOI:** 10.3390/metabo15110746

**Published:** 2025-11-17

**Authors:** Chi-Yi Chen, Che-Yu Hsu, Wei-Pang Chung, Hung-Yu Sun, Tzu-Ching Kao, Tzu-Yi Chen, Xing-Min Li, Wei-Lung Huang, Kung-Chia Young

**Affiliations:** 1 Division of Gastroenterology and Hepatology, Department of Internal Medicine, Ditmanson Medical Foundation Chia-Yi Christian Hospital, Chia Yi City 600, Taiwan; 01290@cych.org.tw; 2 Department of Medical Laboratory Science and Biotechnology, College of Medicine, National Cheng Kung University, Tainan City 701, Taiwan; z11003015@ncku.edu.tw (C.-Y.H.); n168378@mail.hosp.ncku.edu.tw (T.-C.K.); t36124122@gs.ncku.edu.tw (T.-Y.C.); i34129516@gs.ncku.edu.tw (X.-M.L.); 3 Department of Oncology, National Cheng Kung University Hospital, College of Medicine, National Cheng Kung University, Tainan City 701, Taiwan; edgarbun@gs.ncku.edu.tw; 4 Center of Applied Nanomedicine, National Cheng Kung University, Tainan City 701, Taiwan; 5 Department of Physiology, College of Medicine, National Cheng Kung University, Tainan City 701, Taiwan; hysun@gs.ncku.edu.tw; 6 Center of Infectious Disease and Signal Transduction, College of Medicine, National Cheng Kung University, Tainan City 701, Taiwan

**Keywords:** CD63, extracellular vesicles, lean MASLD, metabolic dysfunction-associated steatotic liver disease, nanoparticle tracking analysis, obese MASLD, perilipins

## Abstract

**Background/Objectives**: Metabolic dysfunction-associated steatotic liver disease (MASLD) is highly prevalent worldwide and represents a growing healthcare challenge due to its risk of progression and association with metabolic comorbidities. Extracellular vesicles (EVs), nanosized membrane-bound particles mediating intercellular communication, have emerged as candidate biomarkers in multiple diseases. This study aimed to characterize serum EV profiles in MASLD patients, stratified into obese and lean groups using a body mass index cutoff of 23 for Asians. **Methods**: We enrolled 170 MASLD patients, 83 obese (median age 50, range 20–80) and 87 lean (median age 50, range 20–87), along with 57 non-MASLD controls (median age 44, range 21–86). Serum EV concentrations and particle sizes were quantified using nanoparticle tracking analysis and correlated with clinical and laboratory parameters. EV cargo proteins, including tetraspanins (CD9, CD63) and lipid droplet-associated perilipins (PLIN2, PLIN3), were assessed by Western blotting. **Results**: Obese MASLD patients displayed marked biochemical abnormalities, whereas lean MASLD patients showed levels comparable to non-MASLD controls. Nevertheless, serum EV concentrations were elevated in both the obese and lean MASLD groups. Importantly, in lean MASLD, EV levels correlated strongly with disruptions in lipid and glycemic homeostasis. Furthermore, a reduction in the PLIN3/CD63 ratio was observed in EVs isolated from lean MASLD patients. **Conclusions**: Circulating EVs are elevated in both obese and lean MASLD, but lean patients demonstrate a distinctive decrease in the EV PLIN3/CD63 ratio. These findings highlight the potential of EV profiling to uncover disease heterogeneity and to inform risk stratification in MASLD.

## 1. Introduction

Metabolic dysfunction-associated steatotic liver disease (MASLD) is characterized by hepatic fat accumulation exceeding 5% and is strongly associated with obesity, insulin resistance, type 2 diabetes mellitus (T2DM), and dyslipidemia [1,2,3]. Affecting nearly 25–30% of the global population, its prevalence rises with age [4,5,6]. MASLD can progress from simple steatosis to steatohepatitis, fibrosis, cirrhosis, and hepatocellular carcinoma, while also increasing cardiovascular and extrahepatic cancer risk [3,7]. Resmetirom, a thyroid hormone receptor beta (THR-β) agonist, is the first approved therapy for steatohepatitis with fibrosis, yet improves histology in only 25–30% of patients [8]. With aging populations and unhealthy lifestyles, the healthcare burden of MASLD is expected to grow substantially [5,9].

MASLD occurs in both obese and lean individuals, with prevalence rates of 60–90% and 10–20%, respectively. These subgroups differ in pathophysiology, risk factors, genetic susceptibility, and therapeutic considerations [10,11,12]. In Asian populations, a lower body mass index (BMI) cutoff (<23 kg/m^2^) defines lean MASLD due to a higher incidence of metabolic disorders at lower BMIs compared with Western populations (<25 kg/m^2^) [13,14,15]. Notably, 15–30% of lean Asians develop MASLD, exceeding rates in lean Western populations. While obese MASLD is mainly driven by adiposity-induced insulin resistance, lean MASLD is more influenced by visceral adiposity and genetic predisposition [16,17,18]. Both phenotypes remain vulnerable to progression and complications.

Intracellular lipid droplets (LDs) are lipid storage organelles that serve as energy buffers and protect cells from lipotoxicity; however, their excessive accumulation contributes to hepatic steatosis in MASLD [19]. The LD-resident perilipin (PLIN) family proteins play central roles in regulating the balance between lipid deposit and mobilization, thereby influencing the development of metabolic diseases. Within hepatocytes, PLIN2 stabilizes LDs and prevents premature lipolysis, whereas PLIN3 is primarily involved in the biogenesis and trafficking of nascent LDs [20].

Extracellular vesicles (EVs) are nanoscale, membrane-bound particles actively secreted by cells into the extracellular space. They facilitate intercellular communication by transporting bioactive molecules, thereby modulating cell signaling, coagulation, tissue repair, immune responses, and inflammation [21,22,23,24,25]. EVs are commonly categorized into exosomes, microvesicles, and apoptotic bodies according to their origin and biogenesis [25,26]. Exosomes are generated via the endosome–multivesicular body pathway and released upon fusion with the plasma membrane [24,25,26], whereas microvesicles arise from direct outward budding of the plasma membrane, and apoptotic bodies emerge during programmed cell death through membrane blebbing and fragmentation [25,26]. Alternatively, EVs can also be classified by size into small EVs (100–200 nm) and large EVs (200–1000 nm) [27,28]. Tetraspanins, a family of four-transmembrane-domain proteins, are widely used as canonical surface markers of EVs. Among them, CD9 and CD63 are most frequently applied for EV identification and characterization. Beyond their role as markers, tetraspanins also participate in cargo organization, regulate EV biogenesis, and influence intercellular communication [29,30,31,32,33].

The role of EVs in maintaining physiological homeostasis is increasingly recognized, and their dysregulation has been implicated in diverse pathological conditions [34]. In this study, we examined clinical blood parameters, serum EV profiles, and EV cargo proteins, including the tetraspanins (CD9 and CD63), as well as the hepatic LD-associated perilipins (PLIN2 and PLIN3), in relation to pathophysiological characteristics of obese and lean MASLD compared with non-MASLD controls. Age effects were investigated by stratifying participants into young, middle-aged, and elderly groups.

## 2. Materials and Methods

### 2.1. Patient Samples

MASLD was diagnosed based on hepatic steatosis detected by FibroScan or ultrasound, in combination with either obesity (obese group) or at least two metabolic risk factors (lean group). The metabolic risk factors included increased waist circumference (Asia Pacific criteria, 90 cm for men and 80 cm for women), elevated blood pressure (BP), and abnormal fasting values for triglycerides (TGs), high-density lipoprotein cholesterol (HDL-c), glucose, hemoglobin A1c (HbA1c), homeostasis model assessment of insulin resistance (HOMA-IR), and high-sensitivity C-reactive protein (hsCRP). No significant excess alcohol use was reported (≥30 g/day for men or ≥20 g/day for women), and the exclusion of secondary causes included viral, autoimmune, and drug-induced liver disease.

A total of 170 patients with MASLD were enrolled, comprising 83 obese (median age: 50, range: 20–80) and 87 lean (median age: 50, range: 20–87) subjects. In addition, 57 non-MASLD (median age: 44, range: 21–86) individuals served as controls who was free of hepatic steatosis and T2DM. According to the criteria for Asian populations, a BMI > 23 kg/m^2^ defined obesity, while a BMI < 23 kg/m^2^ defined leanness [35]. Participants were stratified a priori into young (20–39 years; obese MASLD *n* = 30, lean MASLD *n* = 29, controls *n* = 20), middle-aged (40–59 years; obese MASLD *n* = 28, lean MASLD *n* = 29, controls *n* = 20), and older adults (≥60 years; obese MASLD *n* = 25, lean MASLD *n* = 29, controls *n* = 17) based on commonly used WHO and epidemiological classifications.

All participants were recruited at Chiayi Christian Hospital. Written informed consent was obtained from each subject. The study was approved by the hospital’s Institutional Review Board. Serum samples were collected after overnight fasting and stored at −80 °C. Before EV analysis, samples were thawed and centrifuged at 2200 rpm for 3 min to remove protein aggregates, followed by 2000× *g* for 20 min at 4 °C to eliminate cellular debris and contaminants.

### 2.2. Laboratory Assessments

Biochemical parameters in blood samples were analyzed using the routine automated laboratory systems at Chiayi Christian Hospital. In addition, Homeostasis Model Assessment indices, including HOMA-IR (insulin resistance) and HOMA-β (β-cell function), were calculated.

### 2.3. Chemicals, Reagents, and Antibodies

Analytical-grade chemicals used in this study were categorized according to their experimental application. Buffer components comprised methanol (Cat. No. A452-4, Thermo Fisher Scientific, Waltham, MA, USA) and several reagents obtained from VWR/Amresco (VWR, Radnor, PA, USA; AMRESCO, Solon, OH, USA), including sodium chloride (NaCl; Cat. No. 0241-5KG), HEPES (Cat. No. 0485-500G), Tris-HCl (Cat. No. 0234-1KG), glycine (Cat. No. 0167-5KG), sodium dodecyl sulfate (SDS; Cat. No. 0227-1KG), and Tween 20 (Cat. No. PT-0777). Chemicals for preparing protein samples comprised glycerol (Cat. No. 15523, Riedel-de Haën, Seelze, Germany), β-mercaptoethanol (2-Me; Cat. No. 1.15433.0100, Merck, Rahway, NJ, USA), and bromophenol blue (BPB; Cat. No. B8026, Sigma-Aldrich, St. Louis, MO, USA). Chemicals for gel preparation comprised 30% acrylamide solution (Cat. No. BF344-500ML, Protech, Taipei, Taiwan), ammonium persulfate (APS; Cat. No. 215589-100G, Sigma-Aldrich, St. Louis, MO, USA), and N,N,N′,N′-tetramethylethylenediamine (TEMED; Cat. No. TB0508, Bio Basic, Markham, ON, Canada). For Western blot analysis, primary antibodies included anti-CD9 rabbit monoclonal antibody (Cat. No. MA5-31980, Invitrogen, Carlsbad, CA, USA), anti-CD63 mouse monoclonal antibody (Cat. No. GTX28219, Genetex, Irvine, CA, USA), anti-PLIN2 (ADFP) rabbit polyclonal antibody (Cat. No. GTX110204, Genetex, Irvine, CA, USA), and anti-PLIN3 (TIP47) rabbit polyclonal antibody (Cat. No. GTX85492, Genetex, Irvine, CA, USA). Horseradish peroxidase (HRP)-conjugated secondary antibodies consisted of goat anti-mouse IgG (H + L) (Cat. No. C04001, Croyez, Taipei, Taiwan) and goat anti-rabbit IgG (H + L) (Cat. No. C04003, Croyez, Taipei, Taiwan).

### 2.4. Serum EV Isolation via Iodixanol Density Gradient Ultracentrifugation

Buffer saline was prepared by dissolving 1.6 g NaCl and 3.12 g HEPES in 200 mL deionized water (pH 7.4) and sterilized by filtration through a 0.22 µm membrane (Cat. No. 4612, Pall, Port Washington, NY, USA). Serum was diluted twofold with buffer saline (580 µL serum plus 580 µL buffer saline) and mixed with 290 µL iodixanol (Cat. No. D1556-250ML, Sigma-Aldrich, St. Louis, MO, USA) to generate the lower-phase solution. The upper-phase solution was prepared by combining 2.55 mL iodixanol with 14.45 mL buffer saline (3:17). Sequentially, 1.35 mL each of the upper and lower phases was layered into a centrifuge tube (Cat. No. 349622, Beckman Coulter, Brea, CA, USA), followed by 100 µL buffer saline. Tubes were balanced and placed in a TLA100.3 rotor (Beckman Coulter, Brea, CA, USA), then centrifuged at 95,000 rpm for 3 h at 16 °C (Optima MAX-XP instrument). Fractions were collected in 450 µL increments to isolate very-low-density lipoprotein in fraction 1 (F1), low-density lipoprotein in F2, high-density lipoprotein in F5, and EVs in F6 [36].

### 2.5. Nanoparticle Tracking Analysis (NTA)

The concentration and size distribution of serum-derived EVs were measured using NTA on a NanoSight LM10-HS system (Malvern Instruments, Malvern, Worcestershire, UK), equipped with a 60 mW 405 nm laser and a high-sensitivity scientific CMOS camera. Samples were diluted in particle-free phosphate-buffered saline (PBS; Cat. No. 10010023, Gibco, Grand Island, NY, USA) to achieve a final concentration of 10^6^–10^9^ particles/mL. Measurements were performed under constant flow at 20 °C. Each sample was recorded in 15 videos of 60 s each, with the camera level set to 14. Data were analyzed using NanoSight NTA software version 3.2.

### 2.6. Western Blot Analysis

Isolated EV samples were lysed by combining equal volumes of RIPA buffer (Cat. No. 20-188, Merck Millipore, Billerica, MA, USA) supplemented with protease and phosphatase inhibitors (Cat. No. FC0070-0001 and FC0050-0001, Bionovus, Kansas City, MO, USA). Protein concentrations were quantified using the SMART BCA Protein Assay Kit (Cat. No. 21071, iNtRON Biotechnology, Seongnam-si, Republic of Korea). For Western blotting, 10 µg of total protein was boiled in 6x sample loading buffer (0.35 M Tris-HCl at pH 6.8, 30% glycerol, 10% SDS, 5% β-mercaptoethanol, and 0.03% bromophenol blue). Protein samples were then loaded onto a 10% SDS-polyacrylamide gel, separated by electrophoresis (SDS-PAGE) at 80–100 V, and transferred to a PVDF membrane (Cat. No. 10600023, Cytiva, Marlborough, MA, USA) at 100 V for 1.5 h. Protein-coated membranes were blocked with 5% non-fat milk in TBST (Tris-buffered saline with 0.1% Tween 20) at room temperature for 1 h, followed by incubation with primary antibodies at 4 °C overnight. After four washes with TBST over 1 h, membranes were incubated with HRP-conjugated secondary antibodies at room temperature for 1 h. Following four additional washes within 1 h, protein signals were detected using an enhanced chemiluminescence (ECL) solution (Cat. No. K-12045-D50, Advansta, San Jose, CA, USA).

### 2.7. Statistical Analysis

Continuous variables are presented as median values with interquartile ranges (IQRs). Group differences were evaluated using the Mann–Whitney U test or the Kruskal–Wallis H test, as the variables did not follow a normal distribution. Associations between selected parameters were assessed using Spearman correlation analysis and are reported with the coefficient (ρ). All statistical analyses were performed using SPSS software (version 19.0; SPSS Inc., Chicago, IL, USA). Statistical significance was defined as *p* < 0.05 (*), *p* < 0.01 (**), and *p* < 0.001 (***).

## 3. Results

### 3.1. Distinct Clinical Examination and Blood Test Profiling in Obese and Lean MASLD

The results of clinical examinations and blood test parameters in non-MASLD controls, obese MASLD, and lean MASLD patients are shown in Table 1. Compared to the non-MASLD controls, obese MASLD patients exhibited significantly higher levels of BMI, waist circumference, systolic blood pressure (SBP), diastolic blood pressure (DBP) and serum levels of glutamate oxaloacetate transaminase (GOT), glutamate pyruvate transaminase (GPT), creatinine, triglycerides (TGs), and HbA1c, while showing decreased levels of HDL-cholesterol (HDL-c) (Table 1, comparison A, all *p* < 0.05). In contrast, lean MASLD patients showed significantly increased waist circumference, SBP, and DBP compared to non-MASLD controls, without significant changes in other parameters (Table 1, comparison B, all *p* < 0.05).

Further comparison between obese and lean MASLD revealed that obese individuals had higher levels of serum uric acid, insulin, HOMA-IR, and HOMA-β and a higher white blood cell (WBC) count (Table 1, comparison C, all *p* < 0.05). These findings suggest that obese MASLD patients exhibited more pronounced impairment in blood lipid and glycemic homeostasis than their lean counterparts.

Next, clinical parameters were analyzed across three age groups: young, middle-aged, and elderly. In non-MASLD controls, there was a progressive increase in waist circumference, SBP, DBP, TG, HbA1c, glucose, and GOT levels from the young to the elderly groups (Table 2, Supplementary Figure S1A–G). Among obese MASLD patients, only HbA1c levels showed a significant age-related increase (Table 2, Supplementary Figure S1E). In lean MASLD patients, SBP, HbA1c, glucose, and GOT levels also increased progressively with age, following a trend similar to that observed in non-MASLD controls (Table 2, Supplementary Figure S1B,E–G).

### 3.2. Elevated Circulating EV Concentrations in Both Lean and Obese MASLD

**Figure 1:**
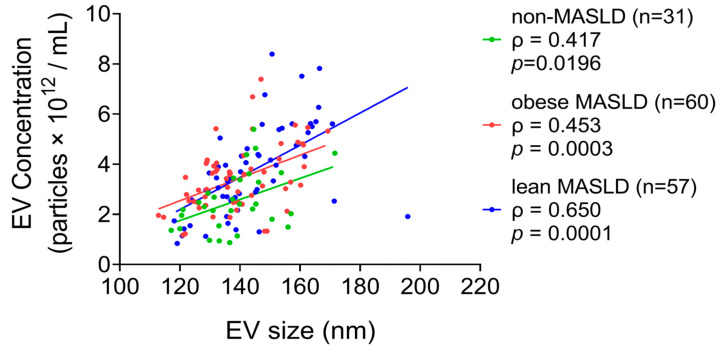
A positive correlation was observed between serum EV particle concentration and mean particle size across all groups.

**Figure 2:**
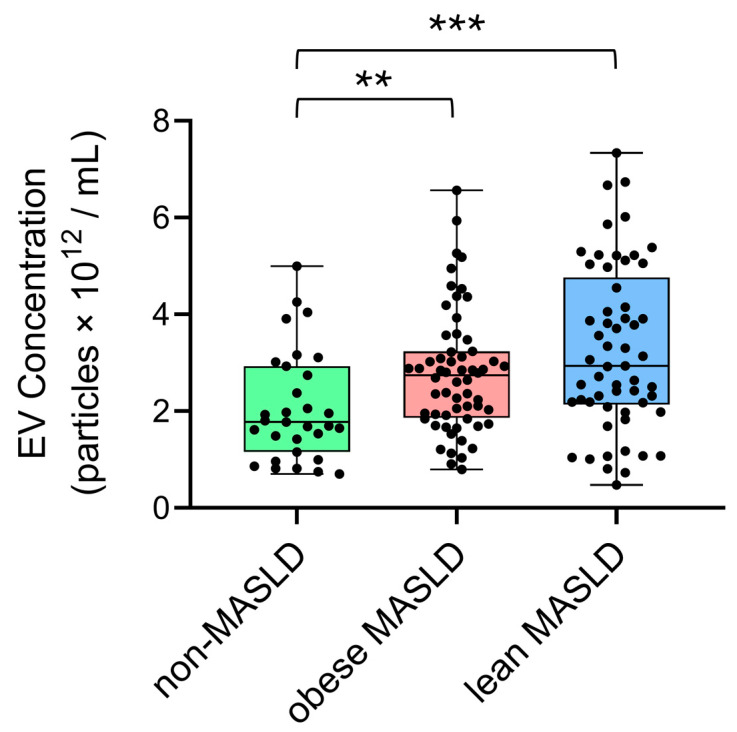
EV concentrations were significantly elevated in obese (*p* = 0.0013) and lean (*p* = 0.0008) MASLD patients relative to non-MASLD controls. Statistical analysis was performed using the Mann–Whitney U test, with significance indicated as follows: *p* < 0.01 (**), and *p* < 0.001 (***).

The concentrations and mean sizes of serum EV particles were positively correlated in all groups, including non-MASLD controls (*n* = 31, ρ = 0.417, *p* = 0.0196), obese MASLD (*n* = 60, ρ = 0.453, *p* = 0.0003), and lean MASLD (*n* = 57, ρ = 0.650, *p* = 0.0001) (Figure 1). Notably, both obese (*p* = 0.0013) and lean MASLD patients (*p* = 0.0008) exhibited significantly higher EV concentrations compared to non-MSFLD controls (Figure 2).

**Figure 3:**
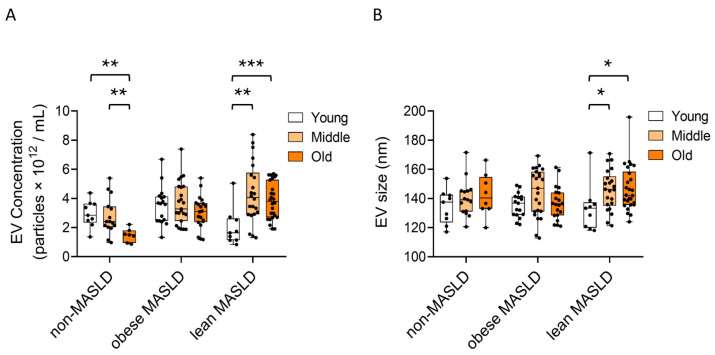
Age-stratified changes in serum EV concentration and size. (**A**) EV concentrations and (**B**) mean EV particle sizes were compared among three age groups in non-MASLD controls and both obese and lean MASLD patients. Statistical analysis was performed using the Mann–Whitney U test, with significance indicated as follows: *p* < 0.05 (*), *p* < 0.01 (**), and *p* < 0.001 (***).

When stratified by age groups, in non-MASLD controls, serum EV concentrations declined with advancing age (*p* < 0.01) (Figure 3A). In contrast, lean MASLD patients showed a significant age-related increase in both EV concentration (*p* = 0.001 and *p* < 0.001) (Figure 3A) and EV size (*p* = 0.023 and *p* = 0.01) (Figure 3B) from the young to the middle-aged and old-aged groups, respectively. This age-related pattern was not observed in either non-MASLD controls or obese MASLD patients.

### 3.3. Correlations Between Serum EV Levels and Lipid Parameters in MASLD

**Figure 4:**
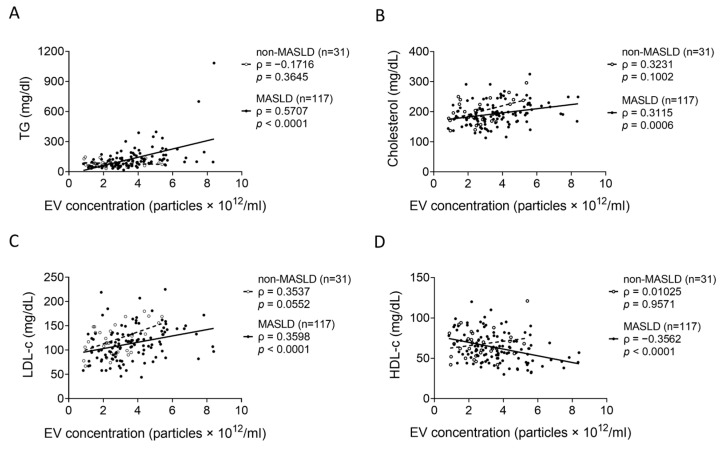
The correlations between EVs and blood tests in non-MASLD and MASLD. The correlations between EV concentrations and blood test parameters: (**A**) TGs, (**B**) cholesterol, (**C**) LDL-c, and (**D**) HDL-c were evaluated using Spearman correlation analysis in non-MASLD controls and all MASLD patients (combined obese and lean).

The associations between serum EV concentrations and blood biochemical parameters were evaluated. In obese and lean MASLD patients combined (*n* = 117), EV concentrations were positively correlated with TGs (ρ = 0.5707, *p* < 0.0001) (Figure 4A), cholesterol (ρ = 0.3115, *p* = 0.0006) (Figure 4B), and LDL-cholesterol (LDL-c) (ρ = 0.3598, *p* < 0.0001) (Figure 4C) but negatively correlated with HDL-c (ρ = −0.3562, *p* < 0.0001) (Figure 4D). None of these correlations were statistically significant in the non-MASLD control group (*n* = 31).

**Figure 5:**
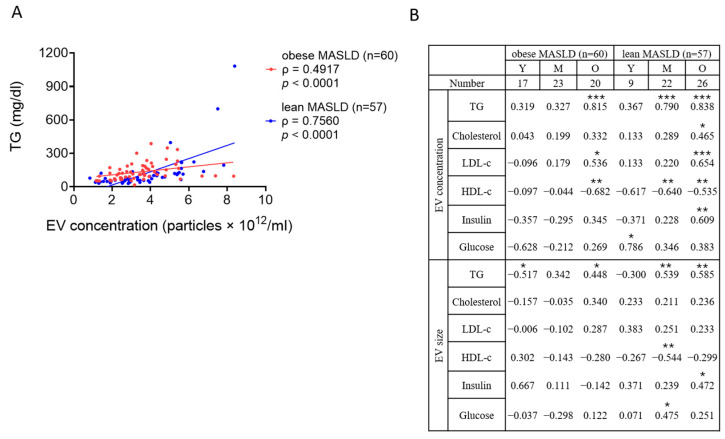
The correlations between EVs and blood tests in obese and lean MASLD. (**A**) EV concentrations and TG levels exhibited positive correlations in obese and lean MASLD patients. (**B**) The table shows the correlations between serum EVs (concentration and size) and blood tests (lipid and sugar) in obese and lean MASLD cohorts, respectively divided into three age groups. Spearman’s ρ values are shown, with the significance levels marked as follows: *p* < 0.05 (*), *p* < 0.01 (**), and *p* < 0.001 (***).

The correlations were further analyzed in obese (*n* = 60) and lean (*n* = 57) MASLD patients. Although TG levels were significantly higher in obese MASLD patients (108.00 mg/dL) compared to lean MASLD patients (76.00 mg/dL) (Table 1, *p* < 0.05), strong positive correlations between EV concentrations and TG were observed in both groups (Figure 5A, *p* < 0.0001), with this association appearing more pronounced in lean MASLD patients (ρ = 0.7560) than in the obese MASLD patients (ρ = 0.4917). Further comparisons stratified by age also revealed that serum lipid and glycemic parameters exhibited stronger and more consistent correlations with both EV concentrations and sizes in lean MASLD patients than in their obese counterparts (Figure 5B).

### 3.4. Decreasing PLIN3/CD63 Ratio in Serum EVs in Lean MASLD

**Figure 6:**
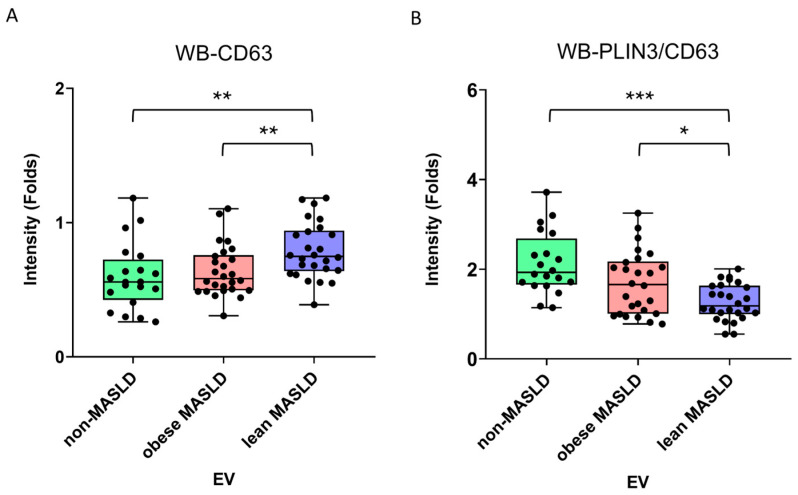
Western blot analysis of serum EV cargo proteins after IDGUC. Lean MASLD patients exhibited (**A**) elevated CD63 levels in serum EVs and (**B**) a reduced PLIN3/CD63 ratio compared with non-MASLD controls and obese MASLD patients. Statistical analysis was performed using the Mann–Whitney U test, with significance indicated as follows: *p* < 0.05 (*), *p* < 0.01 (**), and *p* < 0.001 (***).

Serum samples were fractionated using iodixanol density gradient ultracentrifugation to separate lipoprotein classes and isolate EVs. The fractions were identified as very-low-density lipoproteins (VLDLs, F1), low-density lipoproteins (LDLs, F2), high-density lipoproteins (HDLs, F5), and EVs (F6), validated by the presence of apoB, apoA1, TSG101, and CD63 using Western blotting (Supplementary Figure S2). EV cargo proteins, including the tetraspanins CD9 and CD63, as well as the hepatic lipid droplet-associated perilipins PLIN2 and PLIN3, were subsequently analyzed in F6 samples isolated from clinical serum (Supplementary Figure S3). Notably, PLIN2 and PLIN3 were detected only in the F6 EV fraction, not in the lipoprotein fractions (F1, F2, or F5). Compared with non-MASLD controls and obese MASLD patients, lean MASLD patients exhibited higher CD63 levels in serum EVs, while the PLIN3/CD63 ratio was decreased (Figure 6, Table 3). In contrast, CD9, PLIN2/CD9, PLIN3/CD9, and PLIN2/CD63 ratios were comparable across the three groups (Table 3).

Taken together, the findings suggest that while elevated circulating EV concentrations are generally associated with dyslipidemia in MASLD, the specific metabolic parameters most strongly correlated with EVs might differ between obese and lean phenotypes.

## 4. Discussion

MASLD encompasses a spectrum of liver pathologies driven by excessive fat accumulation and is closely linked to obesity, T2DM, and cardiovascular diseases. Owing to its high prevalence and the rising incidence of insulin resistance, dyslipidemia, and hypertension in modern societies and aging populations, MASLD poses a major challenge to global healthcare systems [3,17,37,38]. While MASLD is most commonly observed in individuals with obesity, lean MASLD represents a distinct phenotype with several unique features. Risk factors for lean MASLD include visceral fat accumulation, genetic variants such as those in patatin-like phospholipase domain-containing 3 (PNPLA3) and transmembrane 6 superfamily member 2 (TM6SF2), as well as the use of steatogenic medications [39,40,41,42]. Compared with obese MASLD, lean patients typically exhibit lower levels of peripheral adiposity, hepatic steatosis, and fibrosis but greater degrees of lobular inflammation and hepatocellular ballooning [40]. Furthermore, lean MASLD has been associated with increased all-cause mortality relative to obese MASLD, as reported in both meta-analyses [43] and long-term cohort studies [10,44,45].

In our study, the lean MASLD group demonstrated serum parameter levels most likely comparable to those of non-MASLD controls. In contrast, the obese MASLD group displayed unfavorable profiles, including elevated GOT, GPT, creatinine, TG, and HbA1c, together with reduced HDL-c levels. These results suggest that blood lipid and glycemic homeostasis is markedly impaired in obese MASLD but remains relatively preserved in lean MASLD. Notably, however, both obese and lean MASLD patients exhibited increased serum EV concentrations compared with non-MASLD controls, indicating that elevated circulating EVs may represent a unifying pathogenic signal of liver stress and a promising surrogate biomarker of hepatic steatosis in MASLD, independent of alterations in conventional biochemical parameters.

Clinical parameters were evaluated across three age groups: young, middle-aged, and elderly. In non-MASLD controls, waist circumference, blood pressure, triglycerides, HbA1c, glucose, and GOT levels progressively increased with age (Table 2, Supplementary Figure S1A–G), indicating that these parameters are influenced by aging even in the absence of hepatic steatosis. Similarly, HbA1c levels showed an age-dependent rise in both obese and lean MASLD patients (Table 2, Supplementary Figure S1E), underscoring the impact of aging on glycemic control in individuals with hepatic steatosis. Notably, in obese MASLD patients, the young group already exhibited elevated TG levels (Supplementary Figure S1D), with no further increase in the middle- or old-age groups, suggesting that TG elevations in obese MASLD are driven more by hepatic steatosis than by aging.

A previous study reported that circulating EV concentrations progressively declined from young to old age in both cross-sectional and longitudinal analyses, whereas EV particle size remained stable by age [46]. This suggests that age-related physiological changes affect serum EV levels. Consistent with this, our study also found an age-dependent decline in EV concentrations among non-MASLD controls, a trend that was absent in obese MASLD patients (Figure 3A). Interestingly, in lean MASLD patients, both EV concentrations and particle sizes were elevated in middle-aged and older individuals compared with the young group (Figure 3A,B), implying that enhanced EV secretion may contribute to age-related pathological processes in lean MASLD.

Hepatic lipid homeostasis is maintained through a balance of fatty acid uptake, de novo lipogenesis, storage in lipid droplets, utilization via β-oxidation, and export through lipoproteins. During fasting, serum TG is primarily derived from hepatic assembly and secretion of very-low-density lipoproteins via the ER–Golgi exocytosis pathway. This process is distinct from the biogenetic mechanisms of EVs, including the endosome–multivesicular body pathway for exosomes, plasma membrane budding for microvesicles, and membrane blebbing and fragmentation for apoptotic bodies [23,24]. Since EV and TG-rich lipoprotein secretion are properly and independently regulated in non-MASLD, no significant correlation between serum EVs and TGs was observed in our controls. In contrast, MASLD patients exhibited a strong positive correlation between serum EV and TG levels, particularly in older obese MASLD patients and in middle-aged and older lean MASLD patients (Figure 5B), with the association being more pronounced in the lean MASLD group (Figure 5A). Consistent with our findings, a previous study demonstrated that circulating EV numbers correlated strongly with elevated TG levels in individuals with metabolic risk factors, including overweight/obesity, hypertension, dyslipidemia, and impaired glucose metabolism [47]. The regulatory mechanisms linking TG-rich lipoprotein production and EV release in MASLD, however, remain to be elucidated.

In hepatic steatosis, excessive lipid accumulation in the liver may alter EV cargo compositions, which then deliver local and systemic pathogenic signals, suggesting their potential as biomarkers for disease progression in MASLD. Tetraspanins CD9 and CD63 have been recognized as standard EV markers for identification but are increasingly also being recognized as functional regulators [48,49,50]. In this study, the lean MASLD patients exhibited higher EV CD63 and lower PLIN3-to-CD63 ratios (Table 3, Figure 6) than non-MASLD controls and obese MASLD patients. CD63 is mainly localized in late endosomes, lysosomes, and multivesicular bodies, where it can facilitate endosomal/lysosomal trafficking and exosome biogenesis, regulating cargo sorting and intercellular communication [29,30,31,32,33]. Increased CD63 in EVs reflects enhanced exosome secretion, often associated with cellular stress, immune activation, or metabolic disturbance [32,51,52,53,54,55]. Therefore, in lean MASLD, higher CD63^+^ EV levels may indicate more hepatocyte stress and correlate with liver injury, inflammation, or fibrosis progression.

PLIN2 and PLIN3 are lipid droplet coat proteins with distinct metabolic roles. While PLIN2 constitutively stabilizes lipid droplets and promotes neutral lipid storage [56,57,58,59,60], PLIN3 dynamically associates with nascent droplets, participating in their biogenesis, trafficking, and lipid turnover during metabolic adaptation [60,61,62,63,64]. The present study primarily focused on EV profiling rather than mechanistic investigation; however, the observed reduction in the PLIN3/CD63 ratio may reflect altered lipid droplet dynamics and EV biogenesis in lean MASLD. PLIN3 is known to regulate lipid droplet formation and turnover, whereas CD63 serves as a canonical EV marker associated with endosomal membranes. In lean MASLD, metabolic stress in the absence of overt obesity may promote lipid droplet remodeling or impair vesicular trafficking, leading to reduced PLIN3 incorporation into EVs relative to CD63. These alterations may represent a distinct cellular adaptive response to lipid dysregulation characteristic of the lean phenotype. We speculate that the reduction in the PLIN3/CD63 ratio reflects an adaptive or stress-related modulation of EV lipid droplet-associated proteins as hepatic lipid handling becomes dysregulated, possibly preceding overt biochemical abnormalities. The alteration in the PLIN3/CD63 ratio may thus occur during an early or metabolically dysregulated stage of MASLD independent of obesity. Nevertheless, the precise temporal dynamics of this change cannot be determined from the present dataset. Further mechanistic studies are warranted to validate these observations and to elucidate the molecular pathways linking lipid droplet remodeling with EV cargo selection in MASLD.

Although most applications of EVs remain at the research stage, advances in standardization, isolation, and quantification are paving the way toward clinical use. Emerging evidence suggests that EVs have potential as noninvasive biomarkers, disease-monitoring tools, and therapeutic vectors. Recent studies have proposed EVs as diagnostic and prognostic biomarkers in MASLD [65,66,67]. In line with these reports, we observed elevated circulating EVs in both obese and lean MASLD, with a distinct reduction in the PLIN3/CD63 ratio in lean patients. These findings suggest that EV profiles may reflect metabolic heterogeneity beyond overt metabolic abnormalities. Future studies should include multiethnic validation, longitudinal assessment, and methodological standardization to establish EVs as robust clinical biomarkers in MASLD. While the therapeutic modulation of EVs was not explored here, these findings support their translational potential. The EV PLIN3/CD63 ratio may also hold potential for evaluating therapeutic response. Although this study was cross-sectional, the reduction observed in lean MASLD likely reflects modifiable alterations in lipid droplet metabolism and EV biogenesis. As these pathways respond to metabolic improvements, the PLIN3/CD63 ratio may dynamically change with treatment. Future longitudinal studies are needed to validate its utility as a biomarker for therapeutic efficacy and disease monitoring in MASLD. As our cohort was limited to Asian participants in a single center and used Asia-Pacific metabolic criteria, larger, multi-center, or multiethnic further studies are needed to validate and extend these observations for broader clinical application.

## 5. Conclusions

Circulating EVs are elevated in both obese and lean MASLD, with lean patients showing a distinct reduction in the PLIN3/CD63 ratio. These findings suggest EV profiling may aid in identifying disease heterogeneity and provide potential biomarkers for risk stratification in MASLD.

## Figures and Tables

**Table 1:** Comparisons of anthropometric and blood test parameters in MASLD study participants.

	Non-MASLD (*n* = 57)	Obese MASLD (*n* = 83)	Lean MASLD (*n* = 87)	*p*-Value *
Gender (F/M)	42/15	40/43	55/32	
Age (yr)	44.00 (32.00–62.00)	50.00 (38.00–65.00)	49.50 (36.00–65.00)	
BMI (kg/m^2^)	22.17 (20.41–23.87)	26.04 (24.57–28.57)	21.78 (20.22–22.59)	AC
Waist circumference (cm)	79.00 (74.00–85.00)	92.00 (84.00–98.50)	83.00 (80.25–91.25)	ABC
SBP (mmHg)	125.00 (112.00–135.00)	137.50 (126.00–145.00)	140.00 (134.50–144.50)	AB
DBP (mmHg)	75.00 (68.00–80.00)	83.00 (75.00–88.00)	86.00 (76.50–88.00)	AB
GOT (IU/L)	20.00 (17.00–24.00)	23.00 (19.00–28.00)	21.00 (18.00–26.00)	A
GPT (IU/L)	18.00 (13.50–23.50)	22.00 (17.00–34.50)	19.50 (14.50–24.50)	AC
ALP (IU/L)	166.50 (148.50–200.50)	190.00 (158.00–237.00)	186.00 (158.50–233.00)	
T-Bil (mg/dL)	0.595 (0.455–0.780)	0.640 (0.440–0.790)	0.605 (0.425–0.805)	
D-Bil (mg/dL)	0.160 (0.105–0.210)	0.165 (0.120–0.215)	0.160 (0.110–0.205)	
Albumin (g/dL)	4.70 (4.30–4.85)	4.60 (4.50–4.80)	4.70 (4.40–4.80)	
Creatinine (mg/dL)	0.650 (0.600–0.825)	0.780 (0.670–0.920)	0.695 (0.610–0.885)	AC
Uric acid (mg/dL)	5.00 (4.30–5.95)	5.80 (4.75–6.70)	5.10 (4.40–5.80)	C
Cholesterol (mg/dL)	190.00 (169.00–216.00)	184.00 (167.00–207.50)	198.00 (174.00–217.00)	
TG (mg/dL)	79.50 (58.00–103.00)	108.00 (71.50–167.50)	77.00 (50.50–113.50)	AC
HDL-c (mg/dL)	62.00 (57.00–74.00)	56.00 (45.00–65.00)	66.00 (57.00–82.00)	AC
LDL-c (mg/dL)	113.00 (94.00–136.00)	113.00 (95.00–132.00)	115.00 (90.50–134.00)	
HbA1c (%)	5.50 (530–5.80)	5.60 (5.40–5.85)	5.50 (5.30–5.80)	AC
Glucose (mg/dL)	89.00 (81.50–96.50)	91.00 (84.50–97.50)	89.00 (85.00–100.00)	
Insulin (μIU/mL)	4.85 (3.20–7.90)	5.95 (4.60–8.55)	3.80 (2.65–5.70)	C
HOMA-IR	1.00 (0.67–1.74)	1.36 (0.99–1.78)	0.84 (0.57–1.37)	C
HOMA-beta	72.00 (50.40–105.33)	84.96 (61.09–117.14)	51.97 (32.77–77.02)	C
WBC (μL)	6500 (5400–8230)	6800 (5860–8130)	6050 (5240–7250)	C
Lymphocytes (%)	32.60 (24.90–36.70)	33.50 (27.70–39.00)	31.40 (26.65–36.10)	
Neutrophils (%)	57.60 (54.60–66.70)	58.00 (53.10–62.50)	60.90 (53.75–65.50)	
PLT (×1000/μL)	251.00 (224.50–287.00)	243.00 (207.00–289.00)	239.50 (200.00–286.00)	

Data are presented as median (IQR). * Statistical analyses were conducted using the Mann–Whitney U test, revealing significant differences (*p* < 0.05) between (A) non-MASLD and obese MASLD, (B) non-MASLD and lean MASLD, and (C) lean MASLD and obese MASLD.

**Table 2:** Age-stratified differences in physical examination and blood test parameters among MASLD groups.

	Young (20–39 yrs)	Middle Age (40–59 yrs)	Old Age (≥60 yrs)	*p*-Value *
**N** **on-MA** **S** **LD**				
Gender (F/M)	13/7	18/2	11/6	
Age (yr)	28.00 (25.50–32.00)	44.50 (41.50–50.50)	66.00 (62.50–70.00)	<0.001
Waist circumference (cm)	75.50 (73.00–81.50)	80.00 (70.50–84.50)	83.00 (77.75–86.50)	0.033
SBP (mmHg)	119.00 (109.00–122.00)	124.00 (112.00–132.50)	135.00 (130.00–140.00)	<0.001
DBP (mmHg)	70.00 (68.50–75.50)	74.50 (67.50–82.00)	82.50 (76.00–85.00)	0.026
GOT (IU/L)	17.50 (16.00–19.50)	21.50 (16.00–24.00)	23.00 (18.50–26.50)	0.033
TG (mg/dL)	59.00 (43.50–73.00)	77.50 (59.50–98.50)	101.50 (83.00–124.00)	0.001
HbA1c (%)	5.25 (5.10–5.40)	5.50 (5.30–5.65)	5.85 (5.80–6.20)	<0.001
Glucose (mg/dL)	82.50 (79.00–91.00)	86.00 (82.00–88.00)	104.50 (98.50–120.00)	0.002
**O** **bese MA** **S** **LD**				
Gender (F/M)	13/17	14/14	13/12	
Age (yr)	36.00 (28.00–38.00)	51.00 (46.50–56.00)	69.00 (66.00–73.00)	<0.001
ALP (IU/L)	167.00 (142.00–198.00)	210.00 (176.50–254.00)	192.00 (158.00–237.00)	0.009
HbA1c (%)	5.50 (5.40–5.70)	5.65 (5.40–5.85)	5.80 (5.50–6.30)	0.003
**L** **ean MA** **S** **LD**				
Gender (F/M)	20/9	20/9	15/14	
Age (yr)	31.00 (25.00–36.00)	50.00 (46.00–54.00)	69.00 (65.00–75.00)	<0.001
SBP (mmHg)	135.50 (133.00–140.00)	140.00 (134.00–142.00)	143.00 (138.00–154.00)	0.018
GOT (IU/L)	18.50 (17.00–23.00)	23.00 (19.00–27.00)	23.00 (19.00–27.00)	0.011
Albumin (g/dL)	4.80 (4.55–5.05)	4.70 (4.55–4.80)	4.60 (4.35–4.70)	0.012
Cholesterol (mg/dL)	195.50 (176.00–208.00)	213.00 (191.00–227.00)	191.00 (158.00–212.00)	0.024
HbA1c (%)	5.30 (5.10–5.50)	5.50 (5.30–5.80)	5.70 (5.50–5.80)	0.001
Glucose (mg/dL)	86.00 (84.00–90.00)	88.00 (84.50–90.00)	99.00 (87.00–105.00)	0.036

Data are reported as median (IQR). * The Kruskal–Wallis H test was used for statistical analysis.

**Table 3:** Comparisons of serum EV cargo protein levels and ratios in the study population.

	Non-MASLD (*n* = 20)	Obese MASLD (*n* = 26)	Lean MASLD (*n* = 26)	*p*-Value *
CD9	0.65 (0.46–0.98)	0.46 (0.34–0.80)	0.52 (0.39–0.70)	0.231
CD63	0.56 (0.44–0.70)	0.58 (0.50–0.75)	0.75 (0.64–0.93)	0.003
PLIN2/CD9	1.02 (0.75–1.85)	0.81 (0.62–2.48)	1.70 (1.29–1.99)	0.166
PLIN3/CD9	2.73 (0.78–3.47)	1.66 (0.96–3.42)	1.69 (1.32–2.29)	0.993
PLIN2/CD63	1.43 (1.05–2.04)	1.16 (0.55–1.49)	1.15 (0.87–1.32)	0.075
PLIN3/CD63	1.93 (1.67–2.57)	1.66 (1.01–2.15)	1.18 (1.03–1.63)	<0.001

Data are shown as median (IQR). * The statistical analysis was performed using the Kruskal–Wallis H test.

## Data Availability

The original contributions presented in this study are included in the article/Supplementary Material. Further inquiries can be directed to the corresponding author(s).

## Supplementary Materials

The following supporting information can be downloaded at https://www.mdpi.com/article/10.3390/metabo15110746/s1, Figure S1: Age-related variations in clinical parameters across study groups; Figure S2: Serum samples from two healthy controls were fractionated by iodixanol density gradient ultracentrifugation to separate lipoprotein classes and isolate EVs; Figure S3: F6 fractions (10 µg of total protein per sample) isolated from serum were subjected to Western blot analysis to examine EV cargo proteins.

## Abbreviations

The following abbreviations are used in this manuscript:

BMI	body mass index
DBP	diastolic blood pressure
EVs	extracellular vesicles
GOT	glutamate oxaloacetate transaminase
GPT	glutamate pyruvate transaminase
HbA1c	hemoglobin A1c
HDL-c	high-density lipoprotein cholesterol
HOMA-IR	homeostasis model assessment of insulin resistance
HOMA-β	homeostasis model assessment of β-cell function
HRP	horseradish peroxidase
hsCRP	high-sensitivity C-reactive protein
LDL	low-density lipoprotein
LDs	lipid droplets
MASLD	metabolic dysfunction-associated steatotic liver disease
NTA	nanoparticle tracking analysis
PLIN	perilipin
PNPLA3	patatin-like phospholipase domain-containing protein 3
SBP	systolic blood pressure
T2DM	type 2 diabetes mellitus
TG	triglyceride
THR-β	thyroid hormone receptor beta
TM6SF2	transmembrane 6 superfamily member 2
VLDL	very-low-density lipoprotein
WBC	white blood cell
