# Supplementary material for: Serum Extracellular Vesicles as Pathogenetic Signals in Obese and Lean Patients with Metabolic Dysfunction-Associated Steatotic Liver Disease
Source: Metabolites. 2025 Nov 17;15(11):746. doi: 10.3390/metabo15110746 (PMC12654108; doi:10.3390/metabo15110746)
Supplement: Supplementary file 1 [file metabolites-15-00746-s001.zip › metabolites-3935350-supplementary/Figure S1-3_PR/Figure S1_PR.pdf]

Supplementary Figure S1

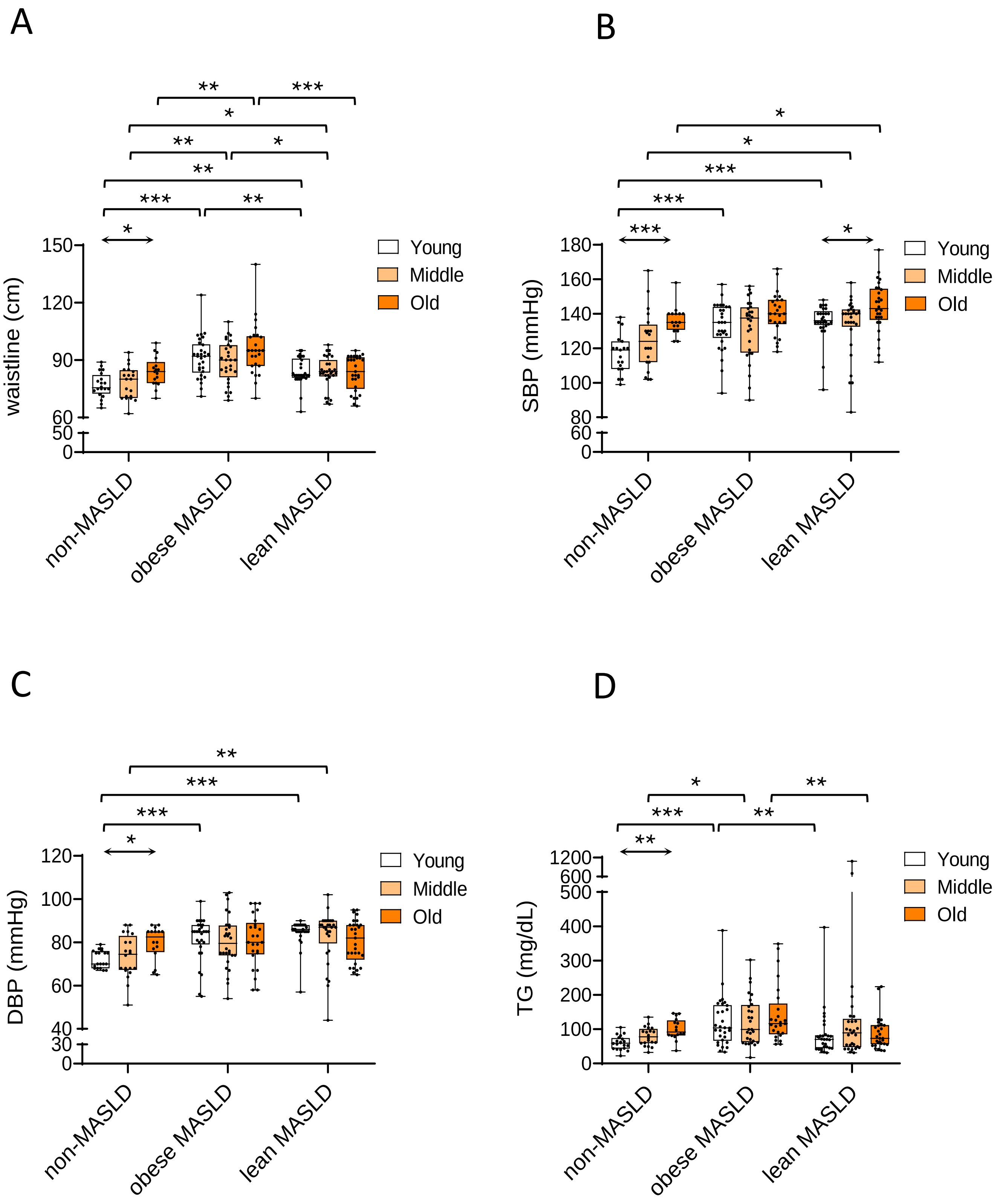

Supplementary Figure S1

E

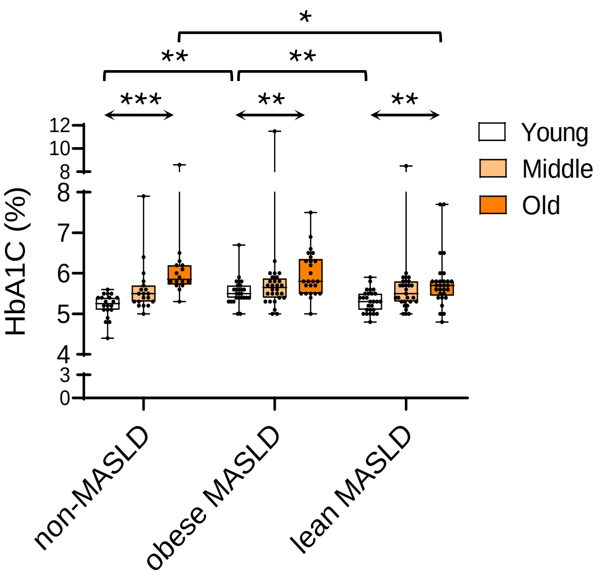

F

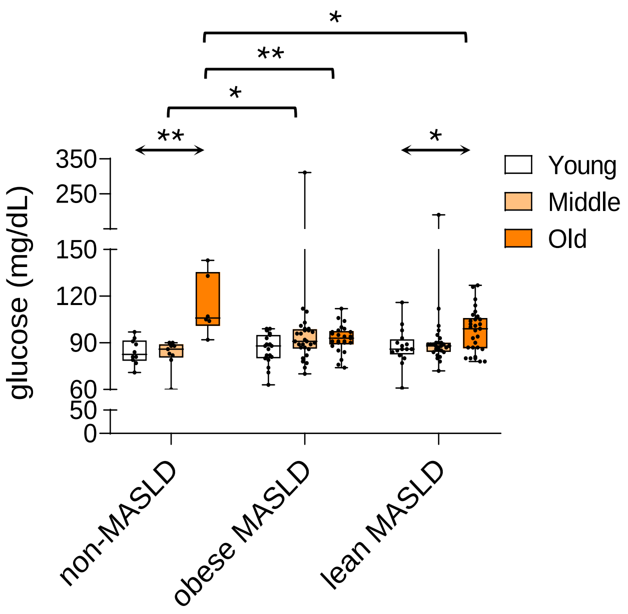

G

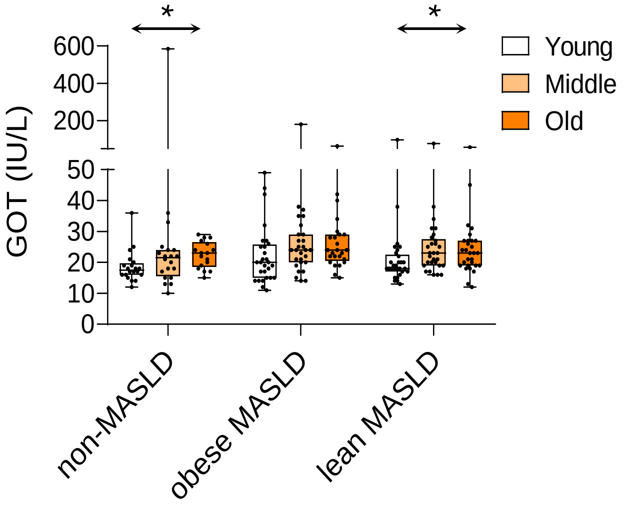

Supplementary Figure S1

Supplementary Figure S1. Age-related variations in clinical parameters across study groups. The physical examination and blood test parameters were compared across three age strata, including young, middle, and old ages, among non-MASLD controls, obese MFSLD and lean MASLD patients. The examined variables encompassed (**A**) waistline, (**B**) SBP, (**C**) DBP, (**D**) TG, (**E**) HbA1c, (**F**) glucose, and (**G**) GOT. Statistical analysis was performed using either the Mann-Whitney U test between 2 groups or the Kruskal-Wallis H test among 3 groups, with significance indicated as follows: $p < 0.05$ (*), $p < 0.01$ (**), and $p < 0.001$ (***).
