# Supplementary material for: Serum Extracellular Vesicles as Pathogenetic Signals in Obese and Lean Patients with Metabolic Dysfunction-Associated Steatotic Liver Disease
Source: Metabolites. 2025 Nov 17;15(11):746. doi: 10.3390/metabo15110746 (PMC12654108; doi:10.3390/metabo15110746)
Supplement: Supplementary file 1 [file metabolites-15-00746-s001.zip › metabolites-3935350-supplementary/Figure S1-3_PR/Figure S2_PR.pdf]

Supplementary Figure S2

A

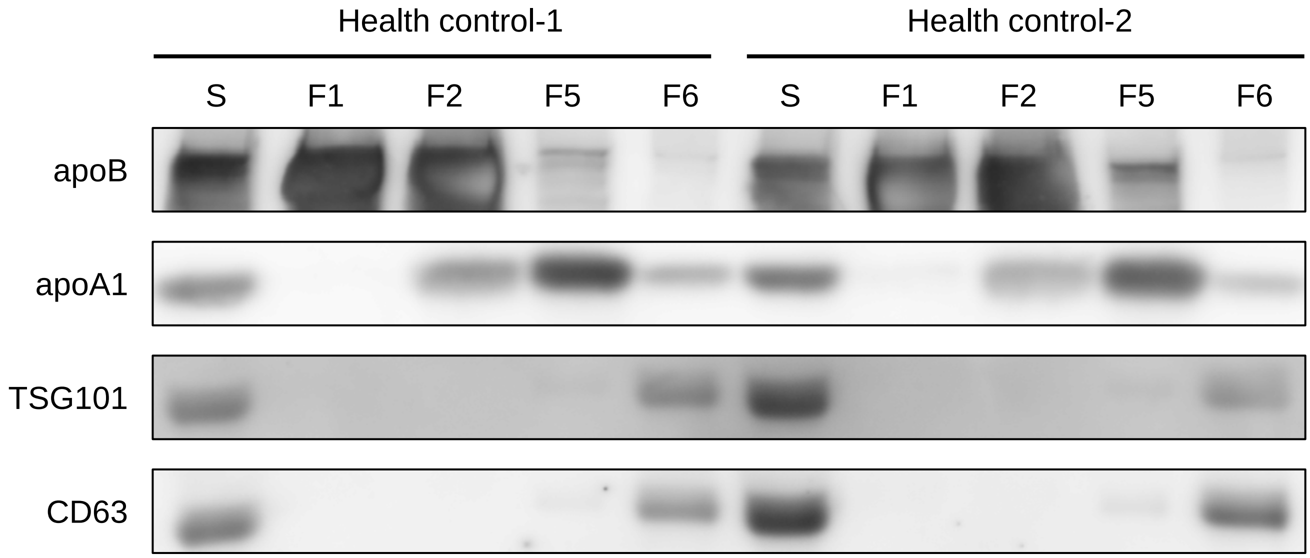

Supplementary Figure S2

B

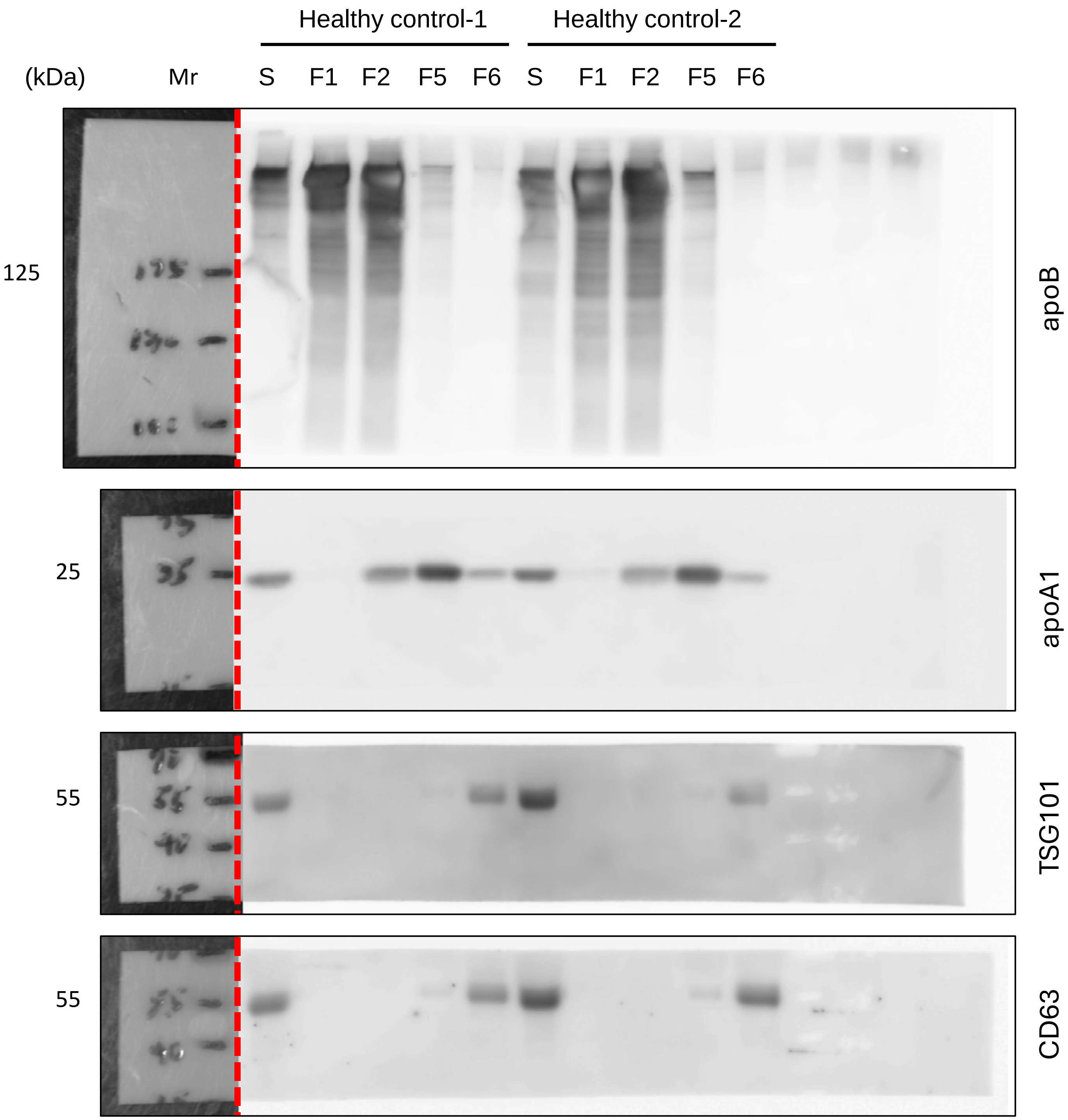

Supplementary Figure S2. Serum samples from two healthy controls were fractionated by iodixanol density gradient ultracentrifugation to separate lipoprotein classes and isolate EVs. The fractions were validated by Western blot analysis using markers including apoB, apoA1, TSG101, and CD63, identifying serum (S), very-low-density lipoproteins (VLDL, F1), low-density lipoproteins (LDL, F2), high-density lipoproteins (HDL, F5), and EVs (F6).
