# Supplementary material for: Serum Extracellular Vesicles as Pathogenetic Signals in Obese and Lean Patients with Metabolic Dysfunction-Associated Steatotic Liver Disease
Source: Metabolites. 2025 Nov 17;15(11):746. doi: 10.3390/metabo15110746 (PMC12654108; doi:10.3390/metabo15110746)
Supplement: Supplementary file 1 [file metabolites-15-00746-s001.zip › metabolites-3935350-supplementary/Figure S1-3_PR/Figure S3_PR.pdf]

Supplementary Figure S3

A

Clinical specimen: Middle age, F6

non-MAFLD

obese MAFLD

lean MAFLD

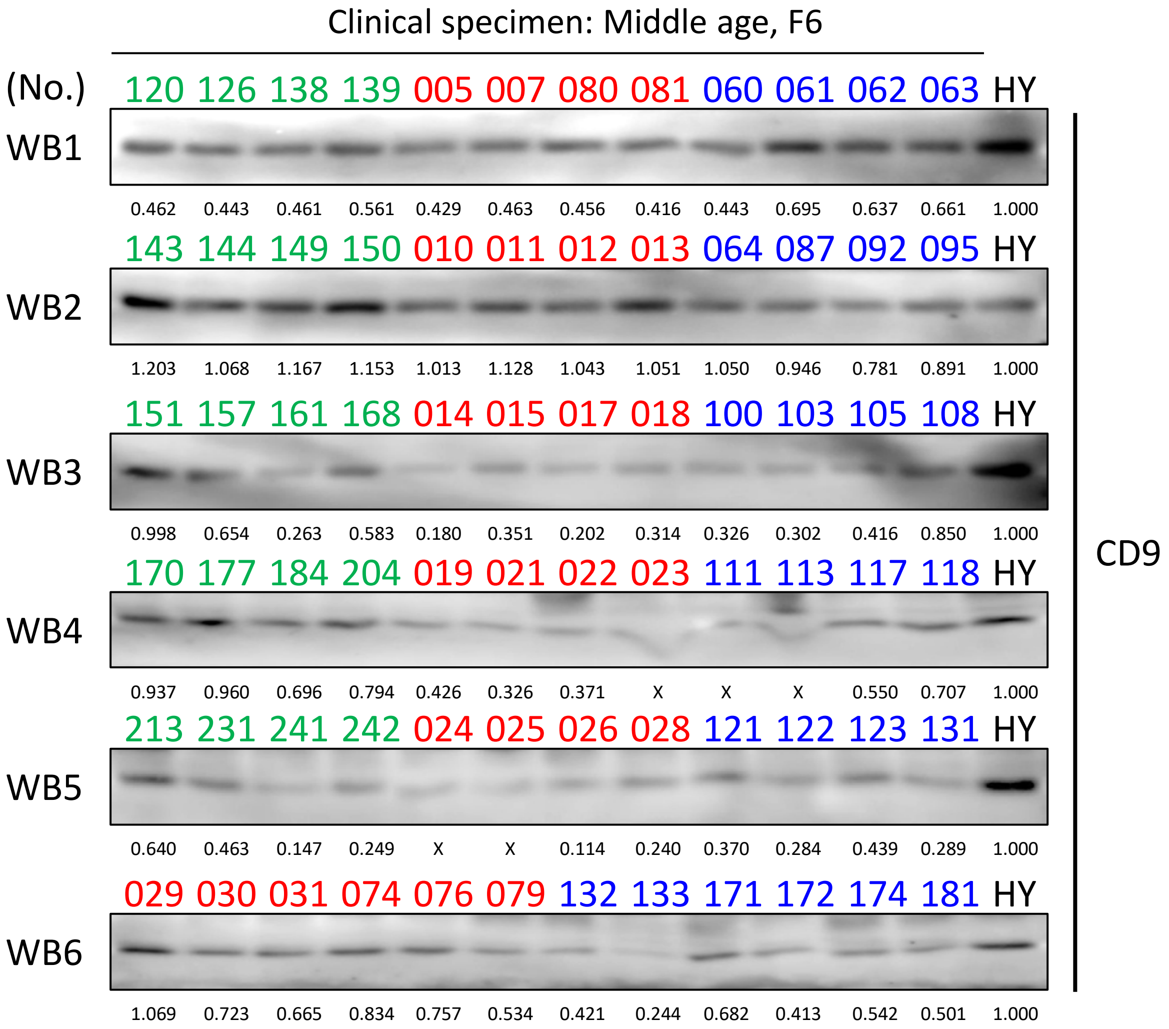

Supplementary Figure S3

B

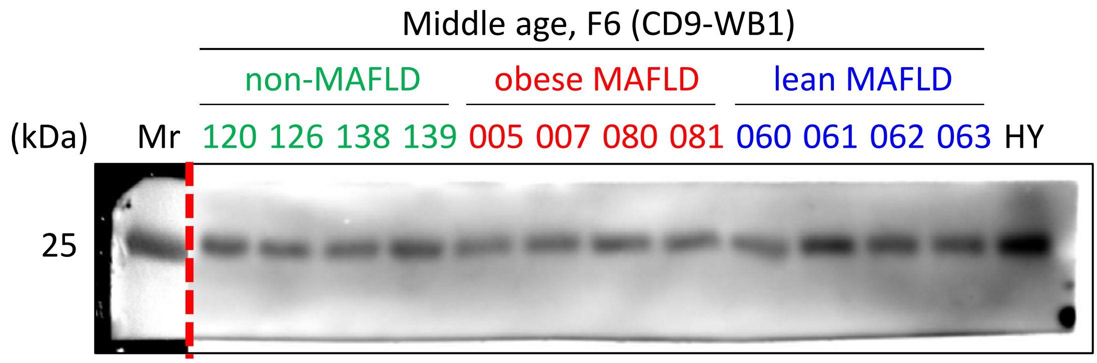

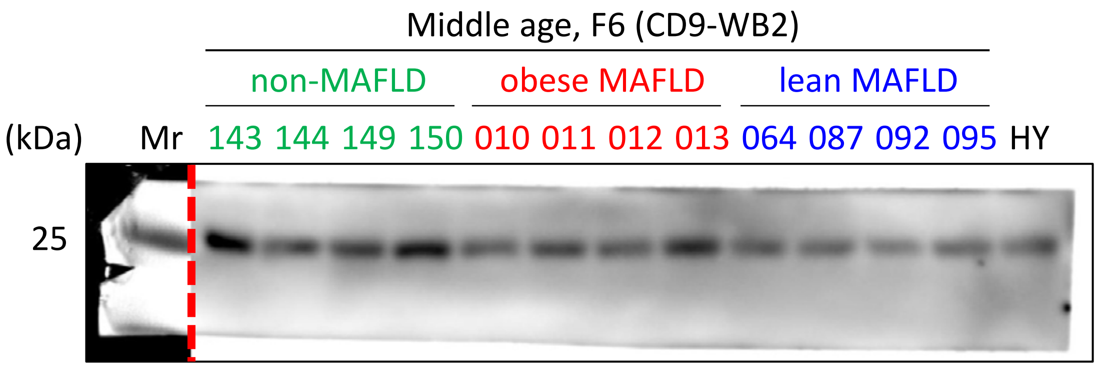

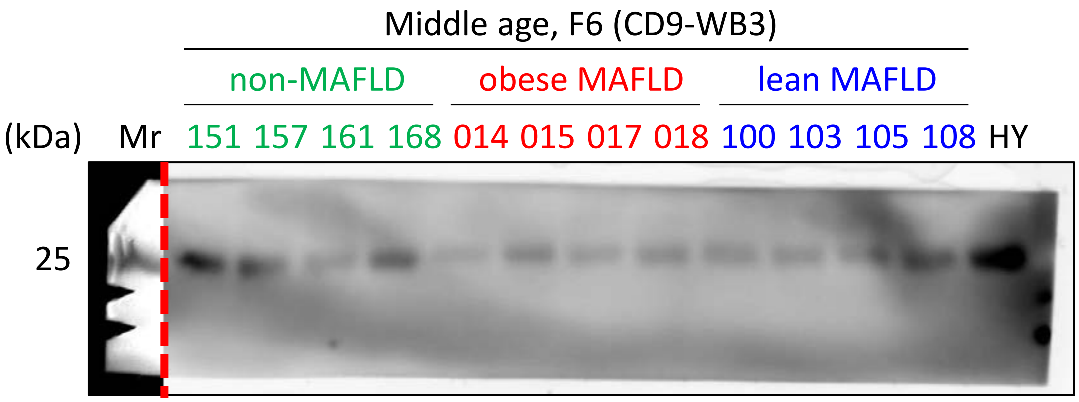

Supplementary Figure S3

C

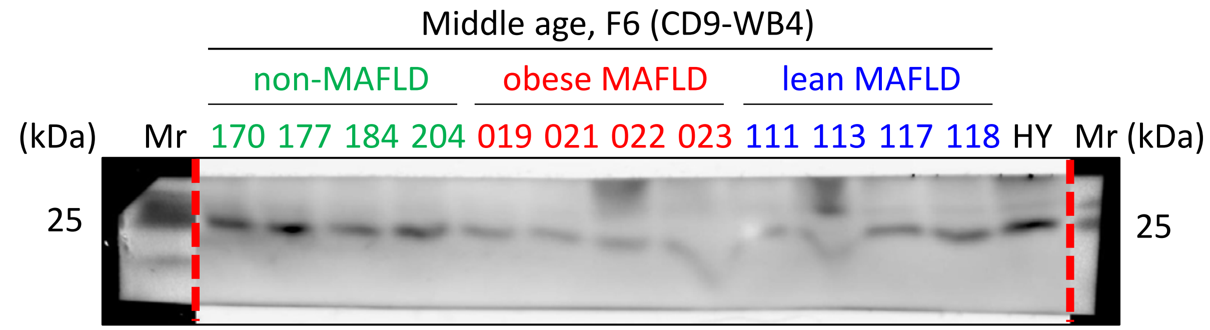

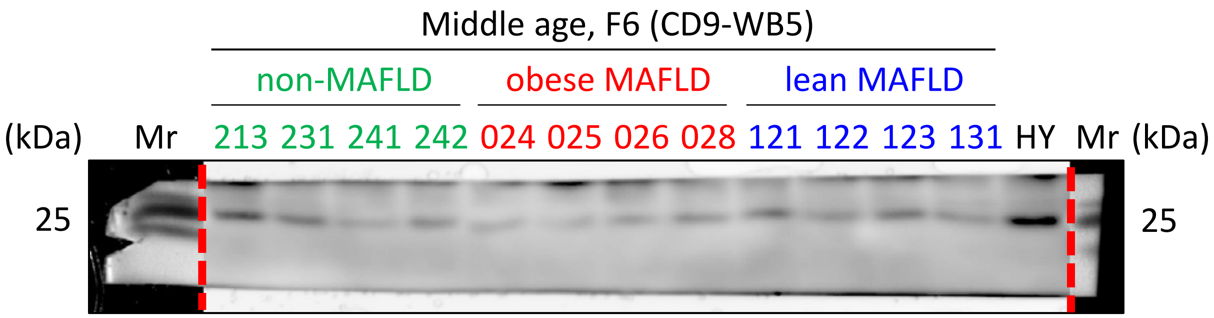

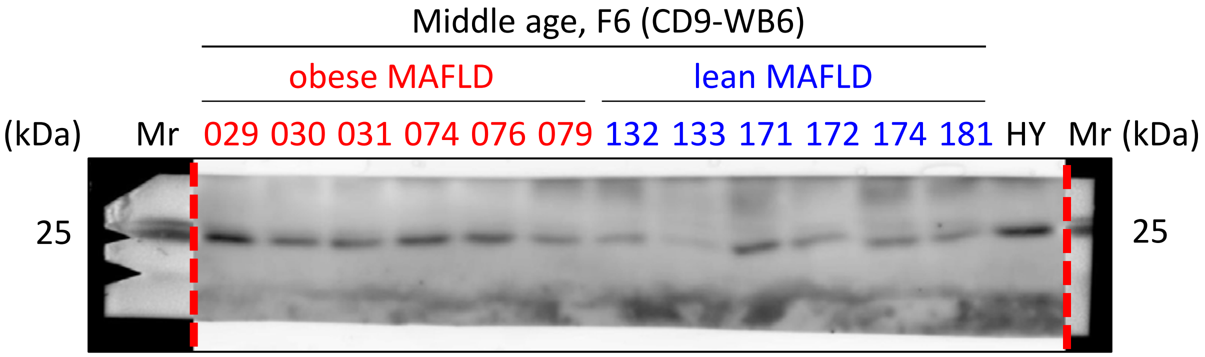

Supplementary Figure S3

D

Clinical specimen: Middle age, F6

non-MAFLD

obese MAFLD

lean MAFLD

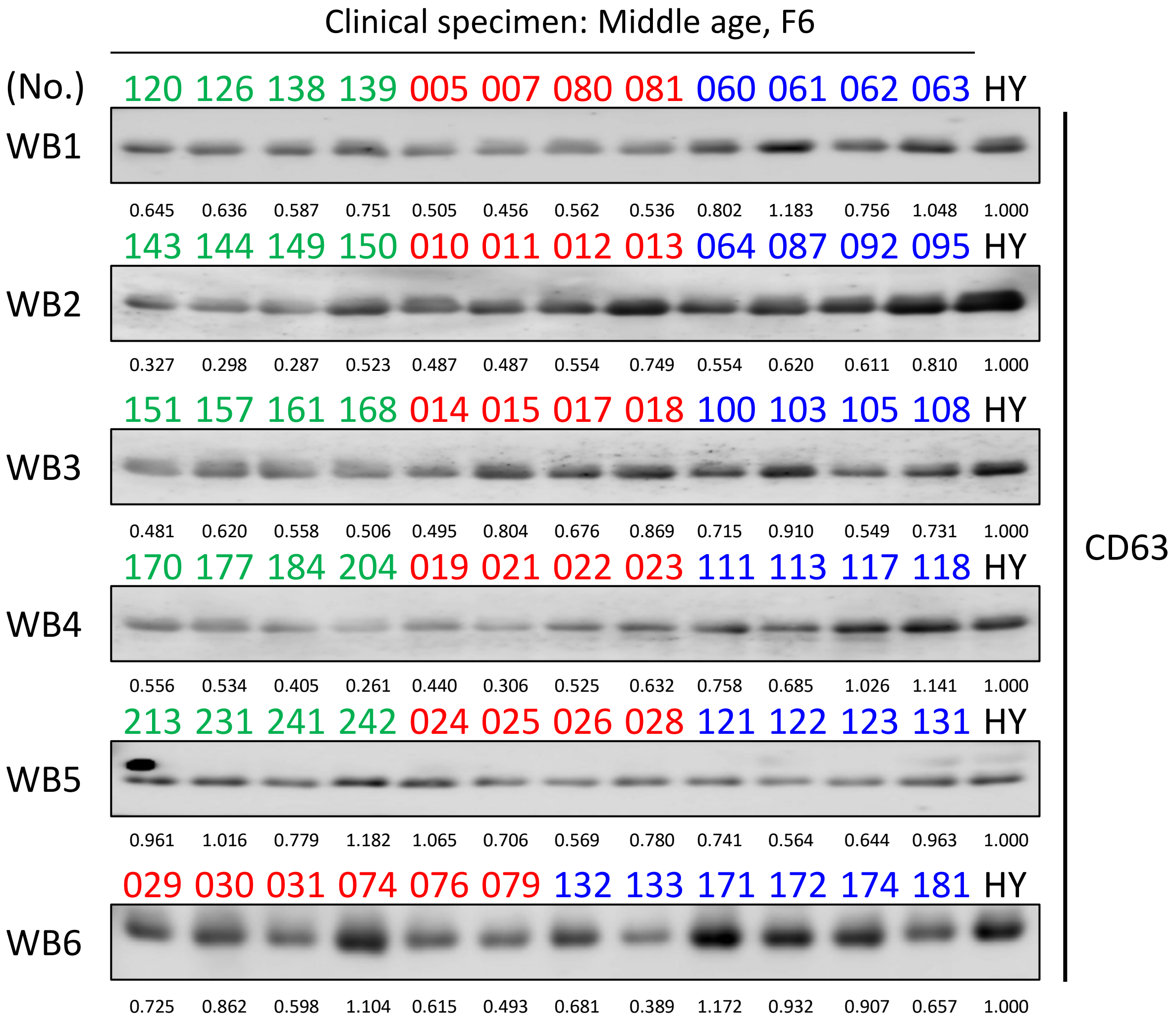

Supplementary Figure S3

E

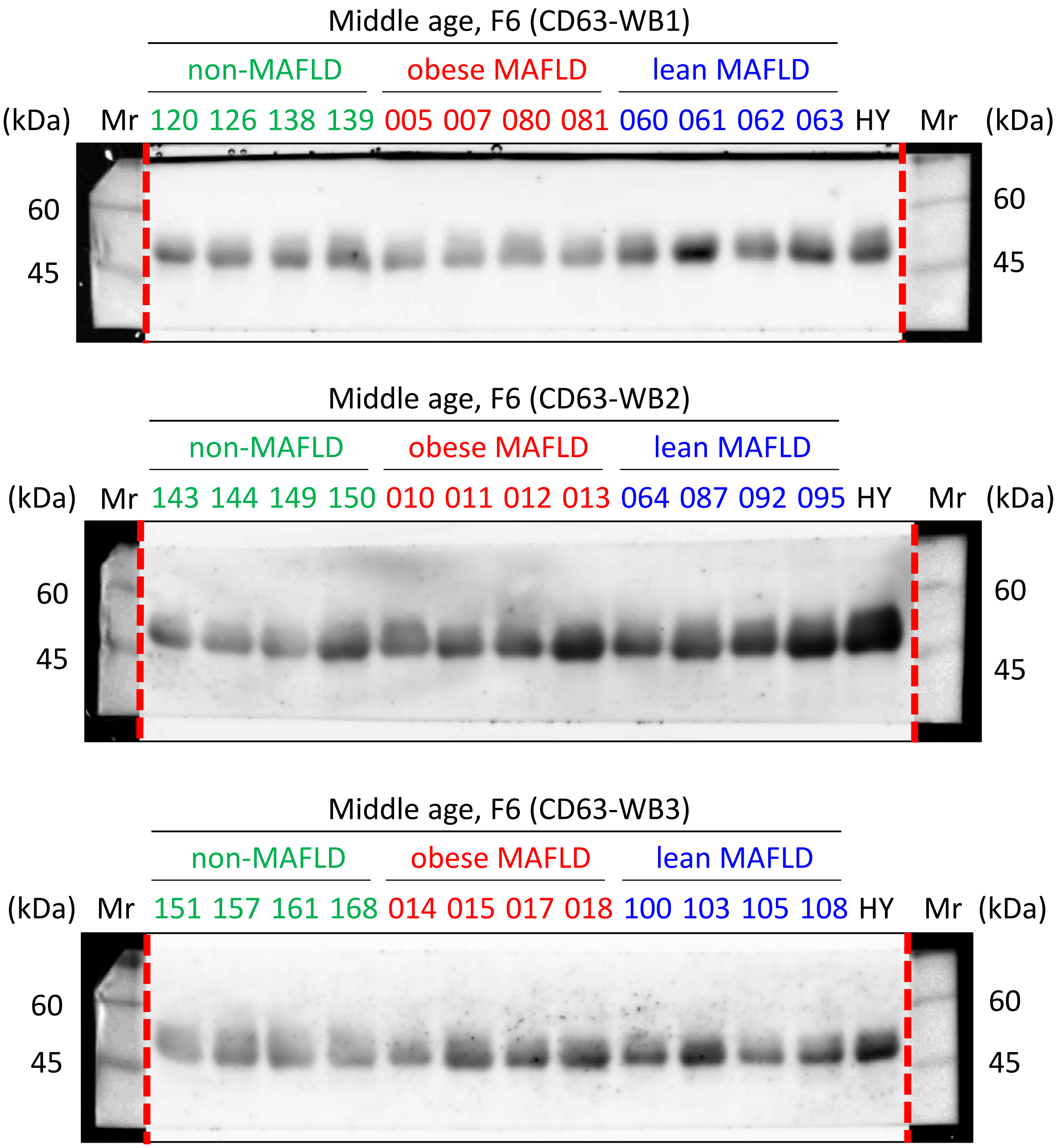

Supplementary Figure S3

F

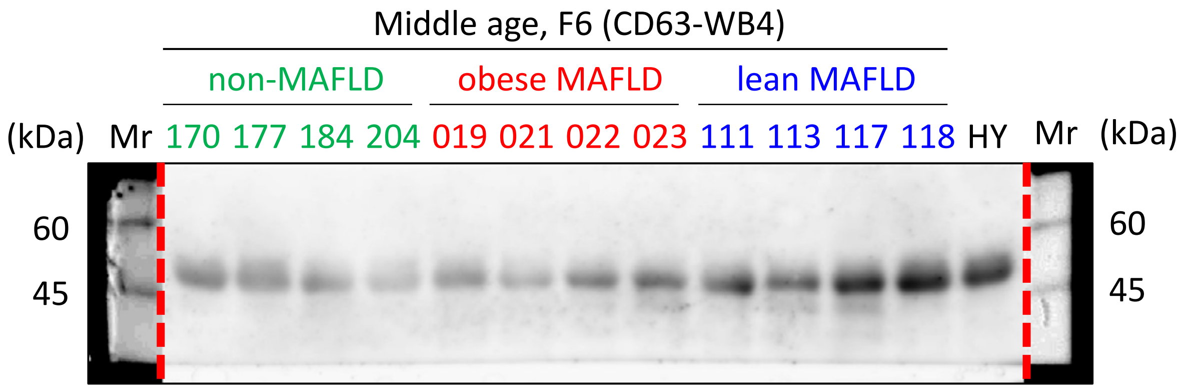

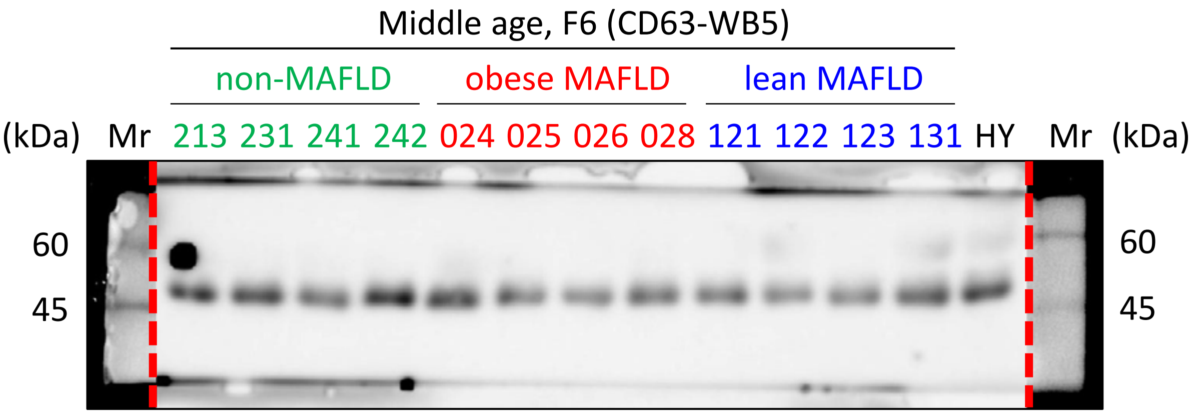

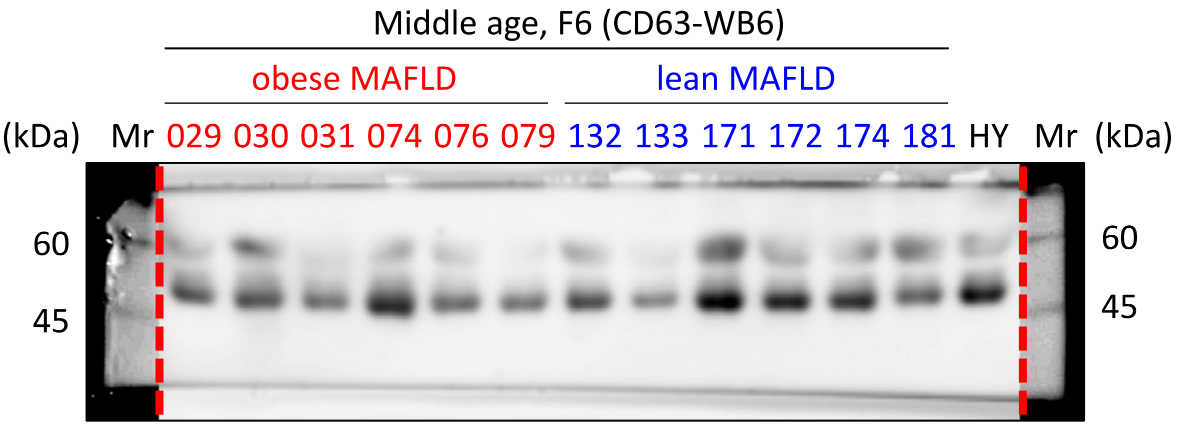

Supplementary Figure S3

G

Clinical specimen: Middle age, F6

non-MAFLD

obese MAFLD

lean MAFLD

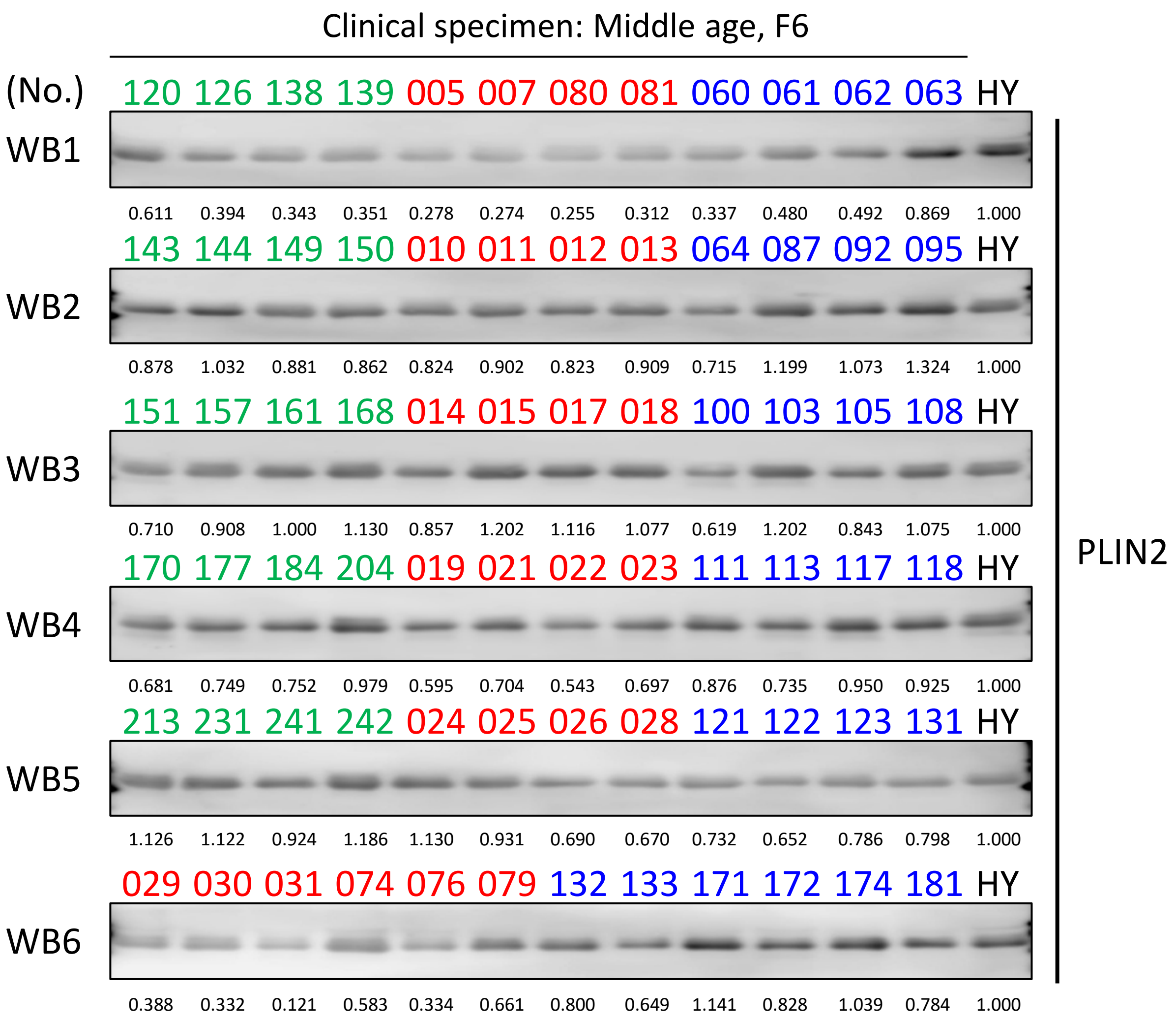

Supplementary Figure S3

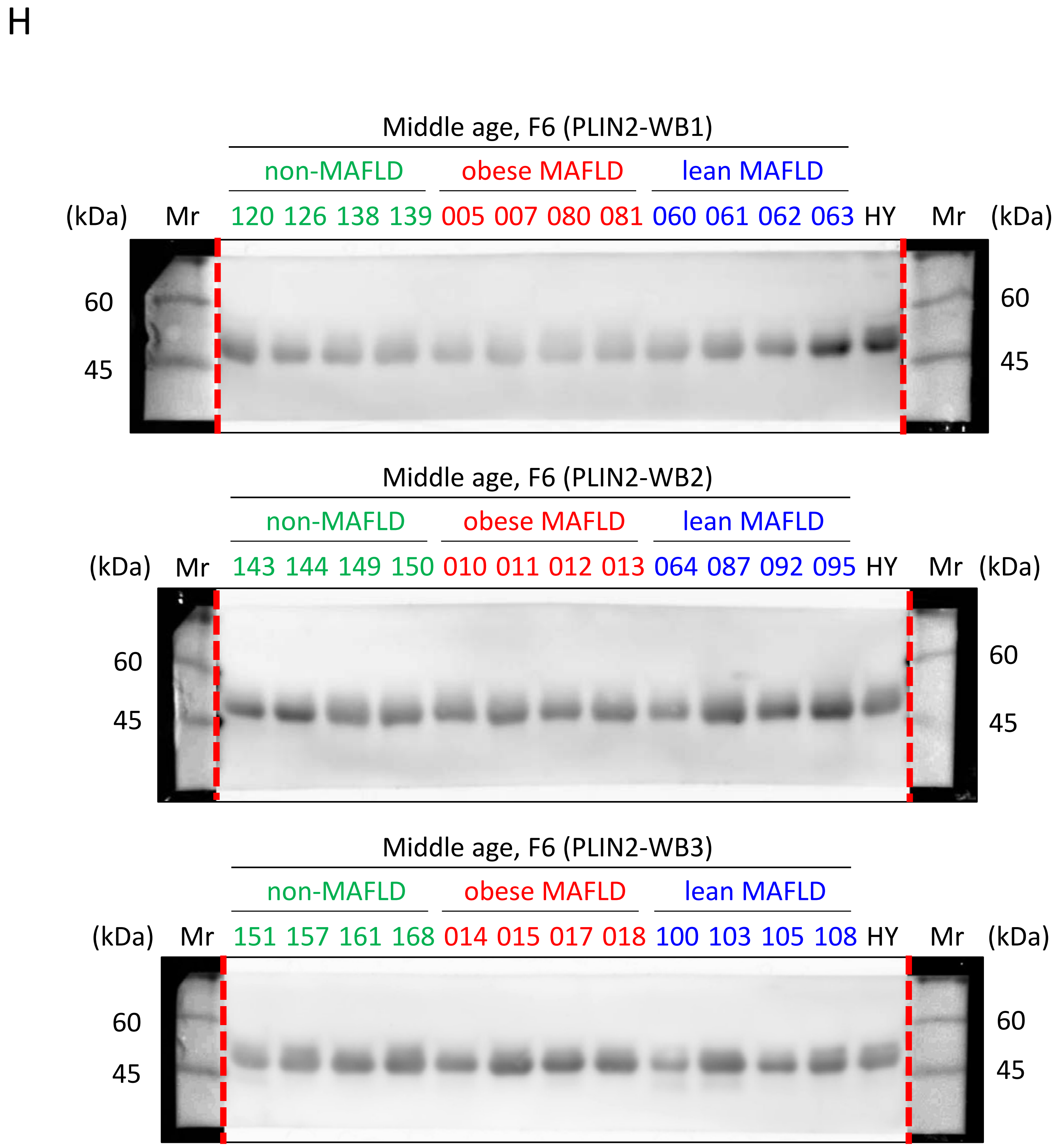

Supplementary Figure S3

I

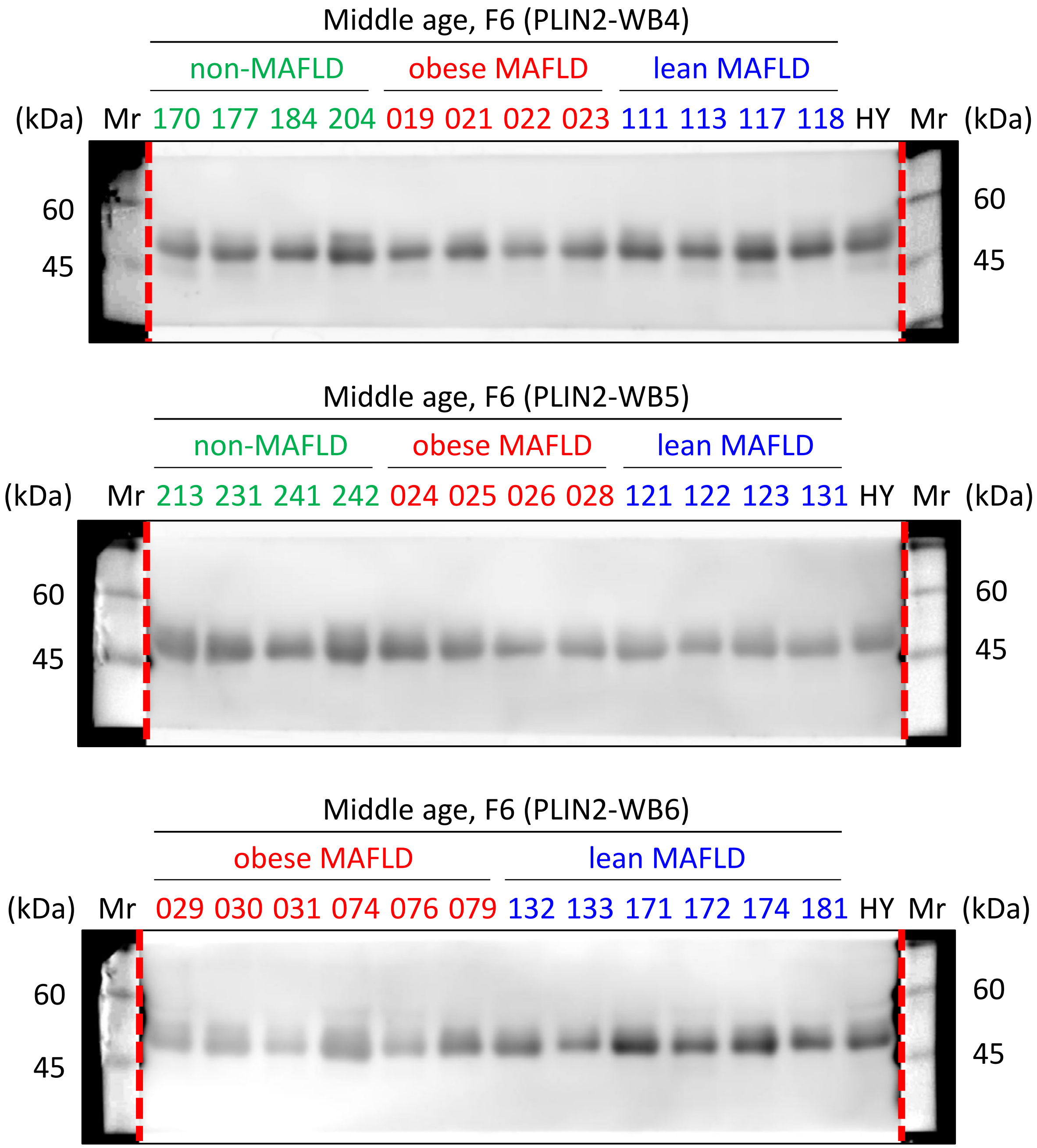

Supplementary Figure S3

J

Clinical specimen: Middle age, F6

non-MAFLD

obese MAFLD

lean MAFLD

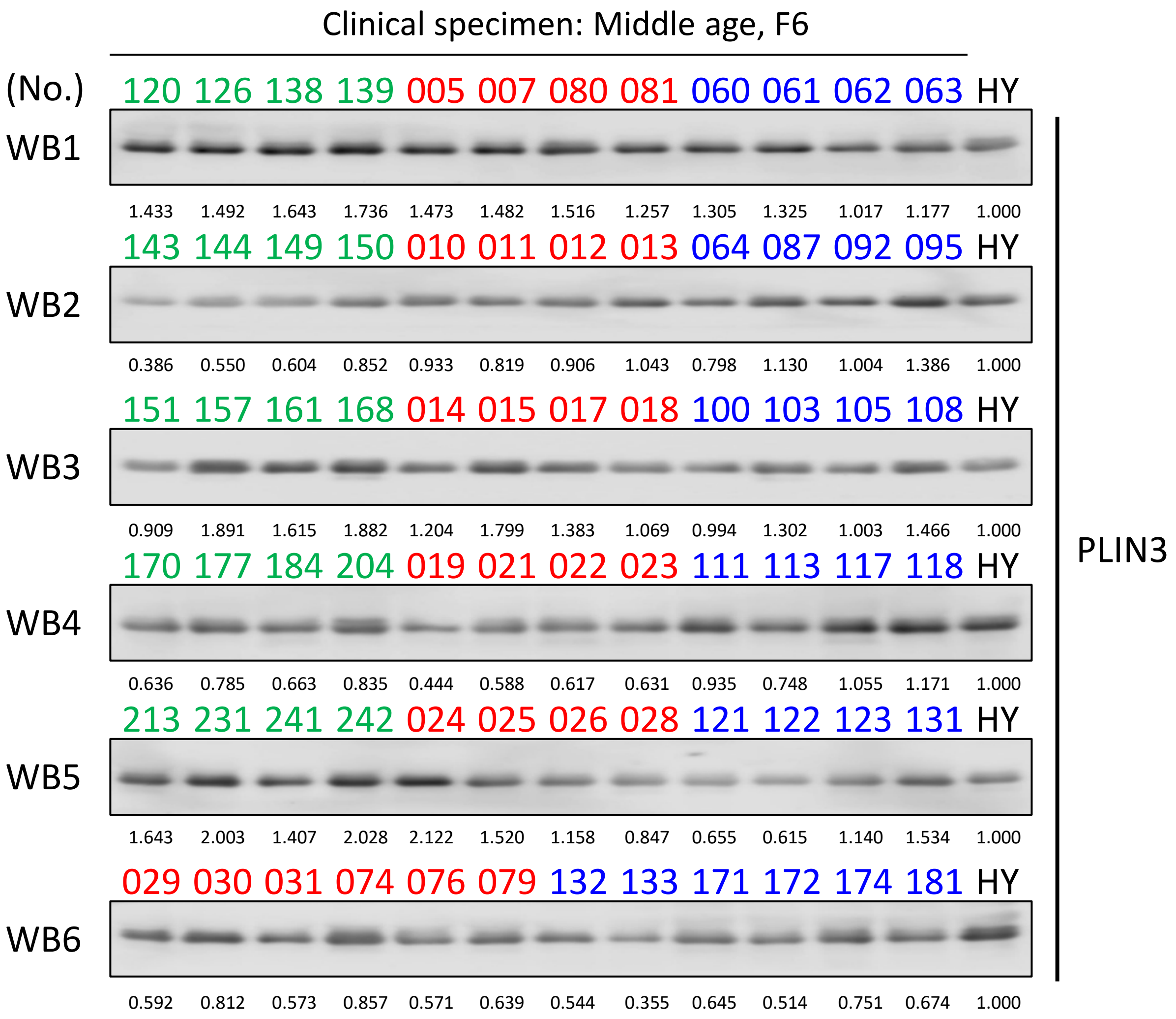

Supplementary Figure S3

K

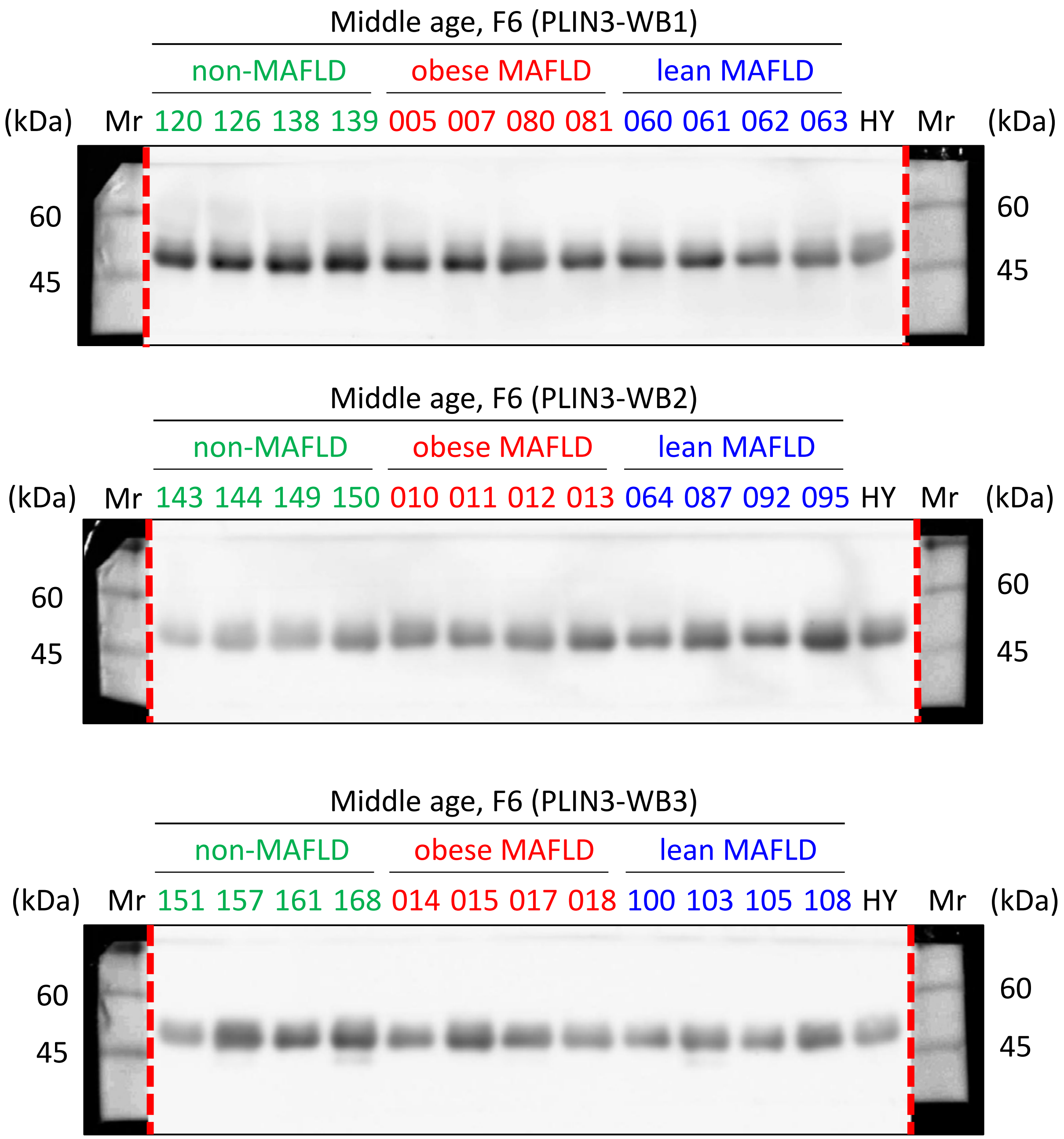

Supplementary Figure S3

L

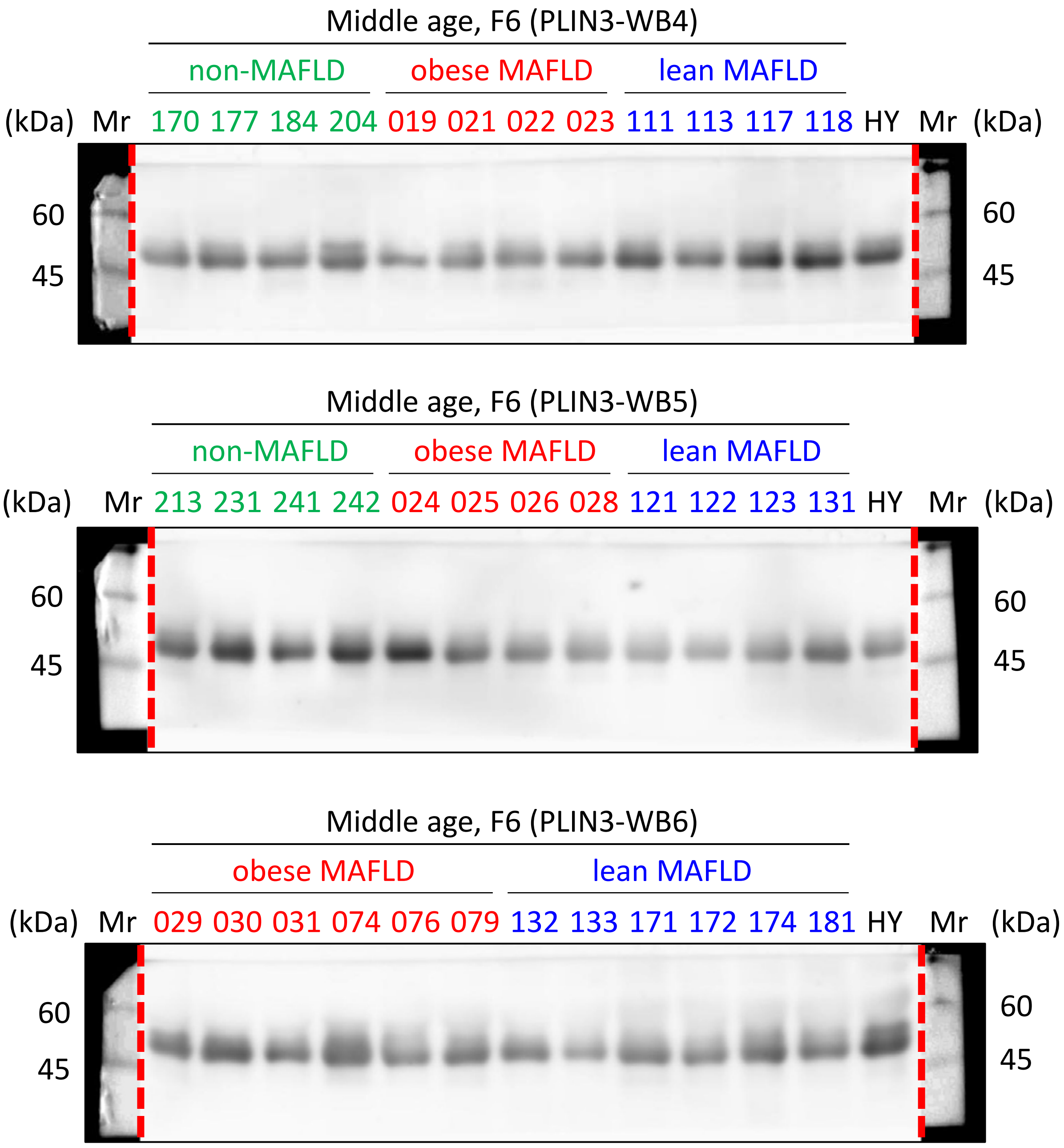

Supplementary Figure S3. F6 fractions (10 µg of total protein per sample) isolated from serum were subjected to Western blot analysis to examine EV cargo proteins. The detected proteins included CD9, CD63, PLIN2, and PLIN3. Western blot quantification and the corresponding original images are presented in the following order: (A–C) CD9, (D–F) CD63, (G–I) PLIN2, and (J–L) PLIN3. The intensity of individual bands was normalized to that of the HY sample within the same Western blot run. Statistical significance in subsequent analyses was assessed as shown in Figure 6 and Table 3.
